# Supplementary material for: The D-Mercator method for the multidimensional hyperbolic embedding of real networks
Source: Nat Commun. 2023 Nov 21;14:7585. doi: 10.1038/s41467-023-43337-5 (PMC10663512; doi:10.1038/s41467-023-43337-5)
Supplement: Supplementary file 1 — Supplementary Information [file 41467_2023_43337_MOESM1_ESM.pdf]

Supplementary Information for
The D-Mercator method for the multidimensional hyperbolic embedding of real networks

Robert Jankowski,[1,2] Antoine Allard,[3,4] Marián Boguñá,[1,2] and M. Ángeles Serrano[1,2,5,*]

[1]*Departament de Física de la Matèria Condensada, Universitat de Barcelona, Martí i Franquès 1, E-08028 Barcelona, Spain*
[2]*Universitat de Barcelona Institute of Complex Systems (UBICS), Universitat de Barcelona, Barcelona, Spain*
[3]*Département de physique, de génie physique et d'optique, Université Laval, Québec (Québec), Canada G1V 0A6*
[4]*Centre interdisciplinaire en modélisation mathématique, Université Laval, Québec (Québec), Canada G1V 0A6*
[5]*ICREA, Passeig Lluís Companys 23, E-08010 Barcelona, Spain*

CONTENTS

I. Differences between Mercator and D-Mercator 2

II. Distribution of angular distances in $\mathbb{S}^D$ model 2

III. Algorithm's time complexity 3

IV. Transformation of unit D-sphere 4

V. Quality of the embeddings of $\mathbb{S}^3$ model 4

VI. Inferring global parameter β 5

VII. Topological properties of embedded synthetic networks 5

VIII. Other topological properties of navigability in the synthetic networks 16

IX. Community concentration of synthetic networks with community structure 18
A. Community overlap 19

X. Topological properties of synthetic networks with community structure 20

XI. Properties of real networks 22

XII. Geometric concentration of real networks 31

XIII. Comparison with topological-based community detection methods 32

Supplementary References 33

* marian.serrano@ub.edu

I. DIFFERENCES BETWEEN MERCATOR AND D-MERCATOR

Mercator is a tool to embed networks in the hyperbolic plane according to the $\mathbb{S}^1$ model. It also applies two methodologies, the model-adjusted machine learning LE technique and the maximum likelihood method. However, there are significant differences between Mercator and D-Mercator.

Nodes' variables. Each node in Mercator is endowed with two variables: a hidden degree κ and an angular position θ in a circle. In D-Mercator, nodes also have hidden degrees; however, they are positioned on the D-sphere and so Mercator stands as a particular case of D-Mercator with $D = 1$. Therefore, we assign a $(D+1)$-dimensional vector to each one. One of the main consequences of this change, purely driven by dimensionality increase, affects the distribution of angular distances. In the $\mathbb{S}^1$ model, this distribution is uniform between 0 and π, yet in higher dimensions, the distribution becomes increasingly peaked around $\Delta\theta = \pi/2$ (see Fig. S1).

No fast version in D-Mercator. Mercator introduced two embedding modes, fast and refined. In the former, the algorithm performs order-preserving adjustment after applying the $\mathbb{S}^1$-modified LE technique. This step allows maintaining larger gaps between communities while readjusting smaller gaps based on the $\mathbb{S}^1$ model. The fast version of Mercator gives already meaningful embeddings. There is no fast mode in D-Mercator, since the readjustment step is not well defined in higher dimensions and it would be as computationally expensive as the ML step, or even more.

II. DISTRIBUTION OF ANGULAR DISTANCES IN $\mathbb{S}^D$ MODEL

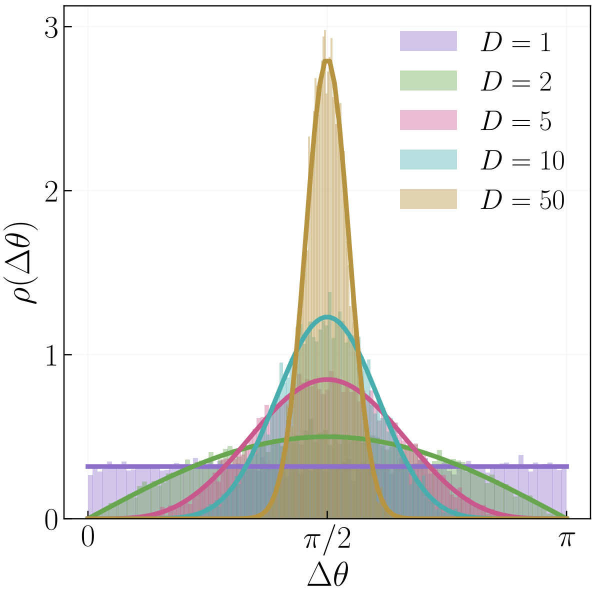

FIG. S1: Distribution of angular distances between pair of nodes for different dimensions. The histograms represent the angular distances between nodes in the synthetic $\mathbb{S}^D$ networks of size 100. Whereas the lines show the analytical solution (Eq. 16 from the main text).

III. ALGORITHM'S TIME COMPLEXITY

We use the Trapezoid Rule to compute the integrals which cannot be solved analytically, e.g., Eq. 13, 17 from the main text. Time complexity is dependent on the resolution, i.e., number of steps, $\mathcal{O}(n)$. Overall, it does not change the final complexity of the D-Mercator which is $\mathcal{O}(N^2)$, where N is the network size. In comparison to the Mercator, the D-Mercator is slower but the time complexity remains the same. The detailed comparison is shown in Figure S2.

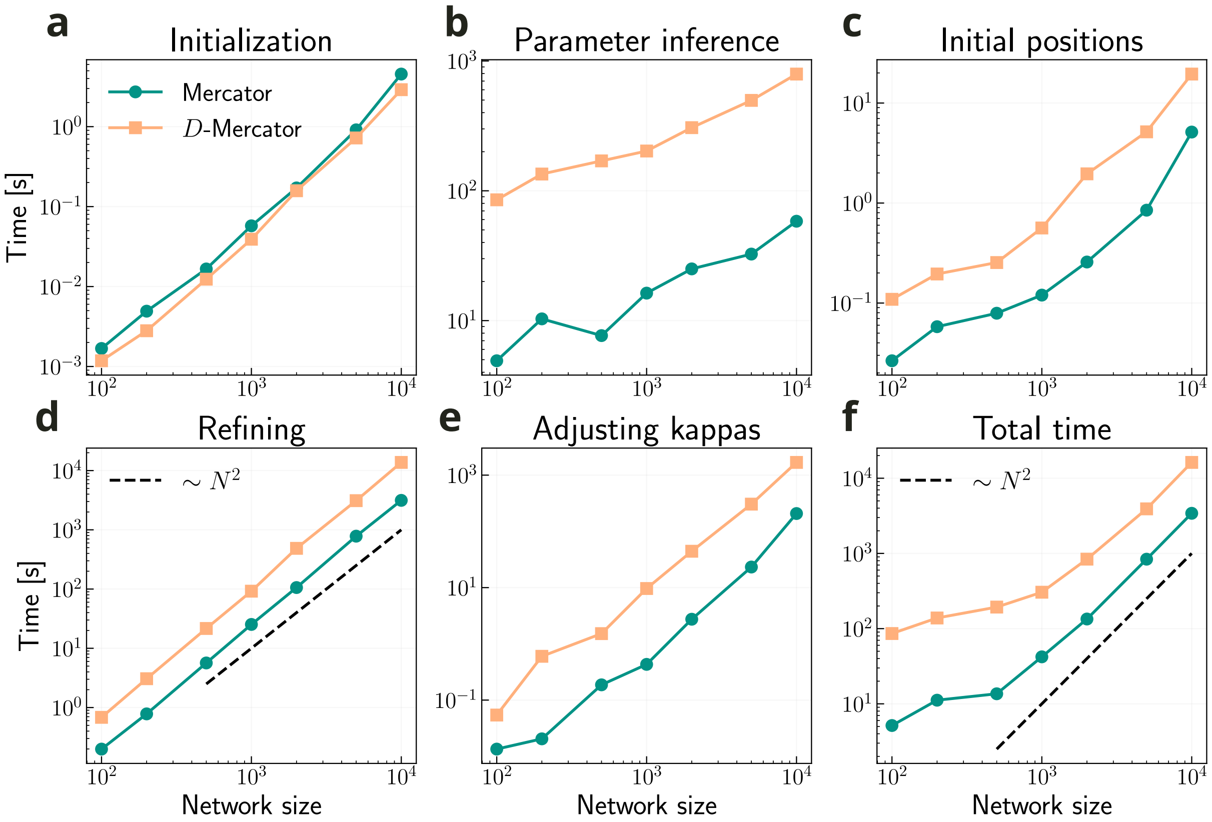

FIG. S2: Comparison of computational complexity between D-Mercator and Mercator in terms of running time versus number of nodes in the network. For Mercator we generated $\mathbb{S}^1$ synthetic network, whereas for D-Mercator $\mathbb{S}^2$ synthetic networks. In both cases, the following parameters were used: $\beta = 2.5D, \gamma = 2.5$. The panel **(a)** shows the initialization part, where all necessary variable are created, in panel **(b)** the time to infer parameter β and set of κ-s is depicted. In panel **(c)** we see the required time to run Laplacian Eigenmaps, whereas in panel **(d)** the maximization likelihood step. The necessary time to run the last step, i.e., adjusting the κ-s is shown in panel **(e)**. Finally, the combined time of the embeddings is presented in panel **(f)**. The results are averaged over 5 realizations. Simulations were conducted on Intel i7-7700K (8 cores, 4.5GHz) with 16GB RAM.

.

IV. TRANSFORMATION OF UNIT D-SPHERE

The procedure of minimizing the distance between the inferred and the real coordinates is shown for $\mathbb{S}^2$ model. However, the extension for higher dimensions is straightforward.

- We start with two sets of coordinates: the generated one (*real coordinates*) and the one from the embeddings (*inferred coordinates*).
- For every node in the network
  1. Rotate the same node i to the axis $[1, 0, 0]$ from both set of coordinates.
  2. Keep the node i fixed and rotate all the rest inferred nodes' coordinates. For each rotated angle compute the average angular distance between the real and inferred positions of the nodes.
  3. Finally, select the node for which average angular distance is minimized. Thus, finding the angle which the inferred nodes' coordinates need to be rotated.
- In some cases, the obtained rotated inferred coordinates might be flipped in the spherical coordinates. Thus, we need to find the rotation angles which maximize the Pearson correlation coefficient between the real and inferred spherical coordinate φ_2.

V. QUALITY OF THE EMBEDDINGS OF $\mathbb{S}^3$ MODEL

Following the procedure shown in the previous section, we tested embeddings of synthetic $\mathbb{S}^3$ models. The obtained Pearson correlation coefficients indicate a great agreement between the real and inferred coordinates.

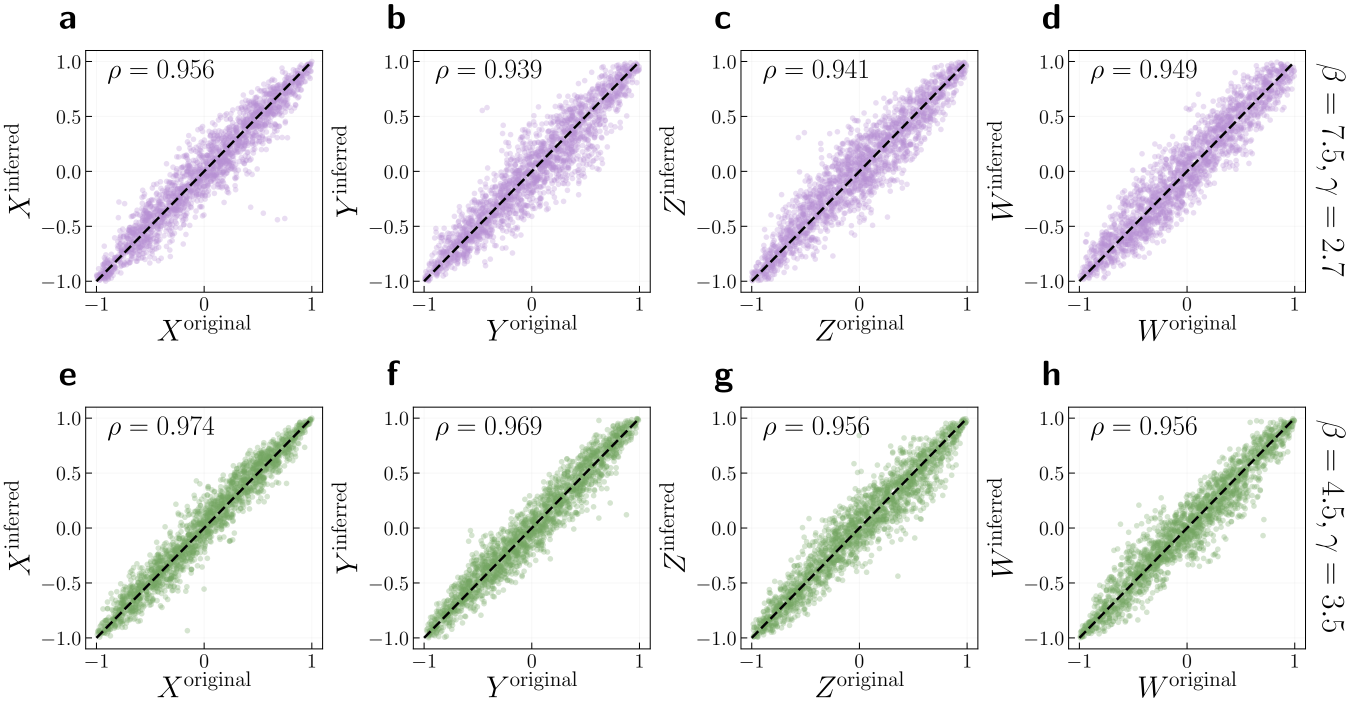

FIG. S3: Relationship between coordinates of the synthetic $\mathbb{S}^3$ networks (*original*) and its embeddings (*inferred*) for network size $N = 2000$. Panels **(a,b,c,d)** show the synthetic network with $\beta = 7.5$ and $\gamma = 2.7$ whereas panels **(e,f,g,h)** depict results for the network with $\beta = 4.5$ and $\gamma = 3.5$. In the top left corner of each figure, the value of the Pearson correlation coefficient between the inferred and original coordinates is reported.

VI. INFERRING GLOBAL PARAMETER β

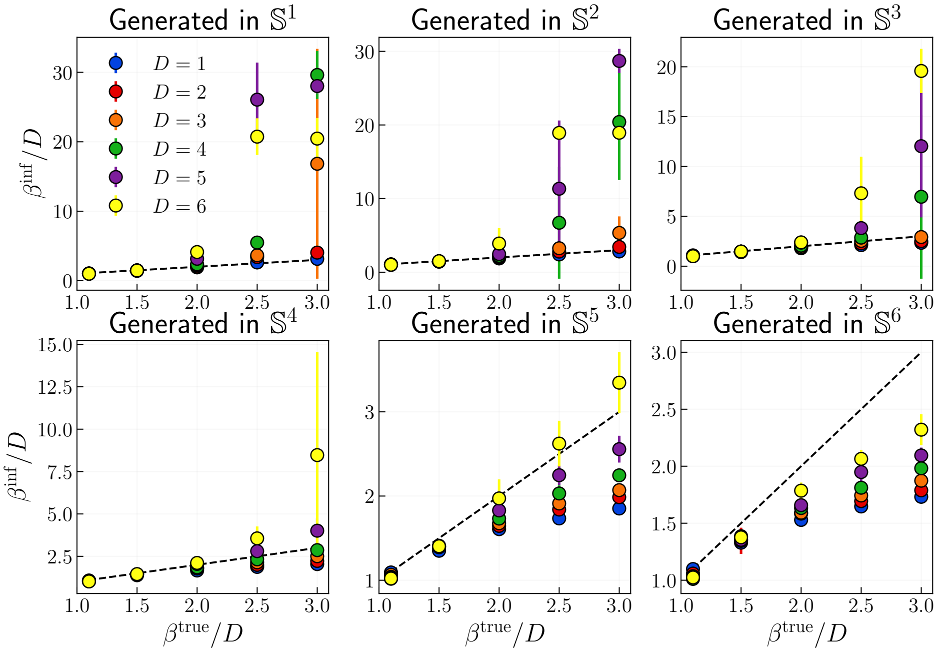

FIG. S4: Relation between the inferred values of β and the generated ones in the synthetic $\mathbb{S}^D$ networks. We generated networks in $D_{\text{in}} = \{1, 2, 3, 4, 5, 6\}$ and embedded them in $D_{\text{out}} = \{1, 2, 3, 4, 5, 6\}$ while changing the value of β. The following parameters were used: $N = 2000, \gamma = 2.7$. Results were averaged over 5 realizations.

VII. TOPOLOGICAL PROPERTIES OF EMBEDDED SYNTHETIC NETWORKS

To test D-Mercator on the synthetic networks more profoundly, we generated the synthetic networks in $D_{\text{in}} = \{1, 2, 3, 4, 5\}$ and embedded them in $D_{\text{out}} = \{1, 2, 3, 4, 5\}$ while focusing on the topological properties of the networks obtained from the embeddings. In the experiments we used: $N = 2000$, $\gamma = 2.1$ or 2.7 and $\beta = 1.5D$ or $2.5D$. Figures S5–S14 depict the topological validation of the embeddings.

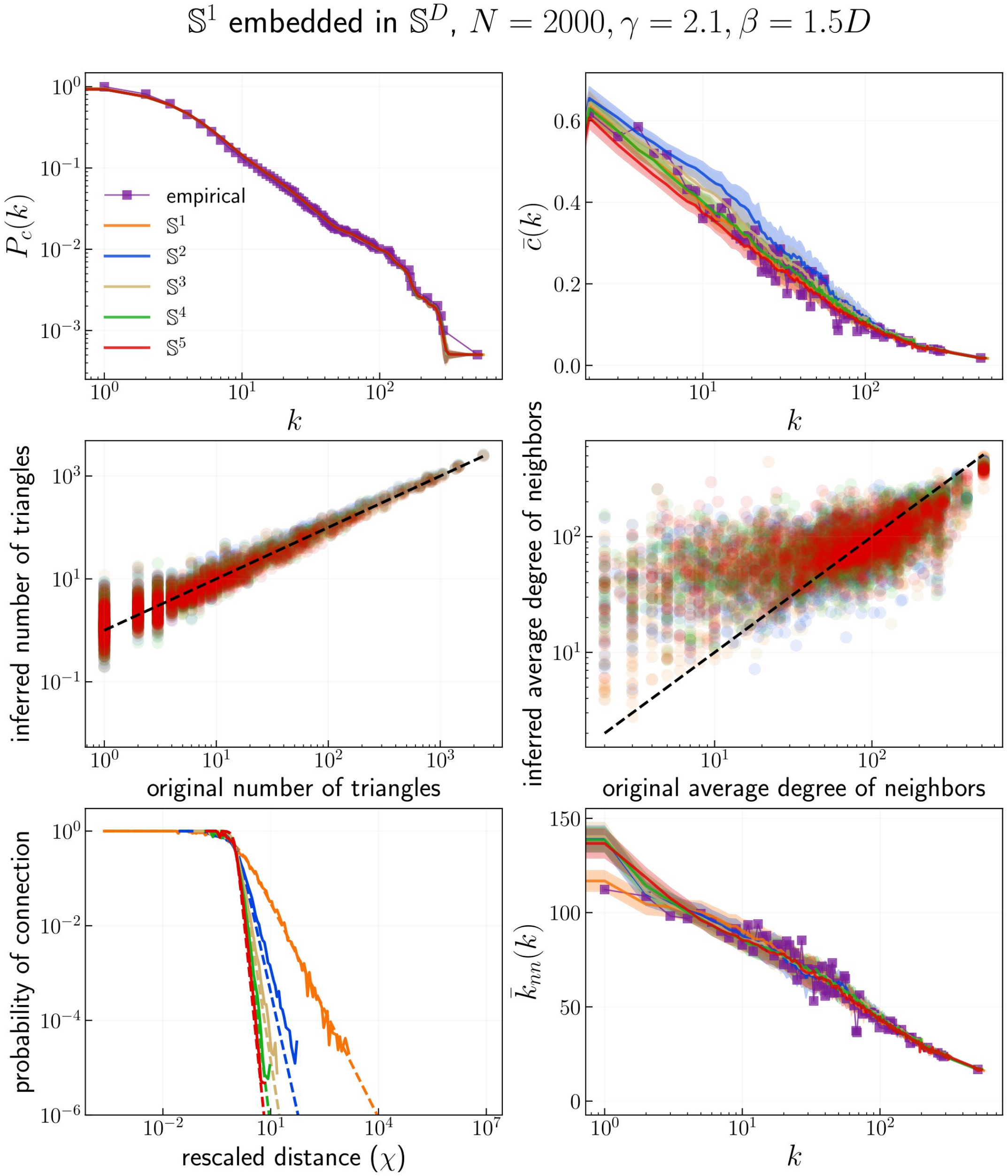

FIG. S5: Topological validation of the embeddings of the $\mathbb{S}^1$ model.

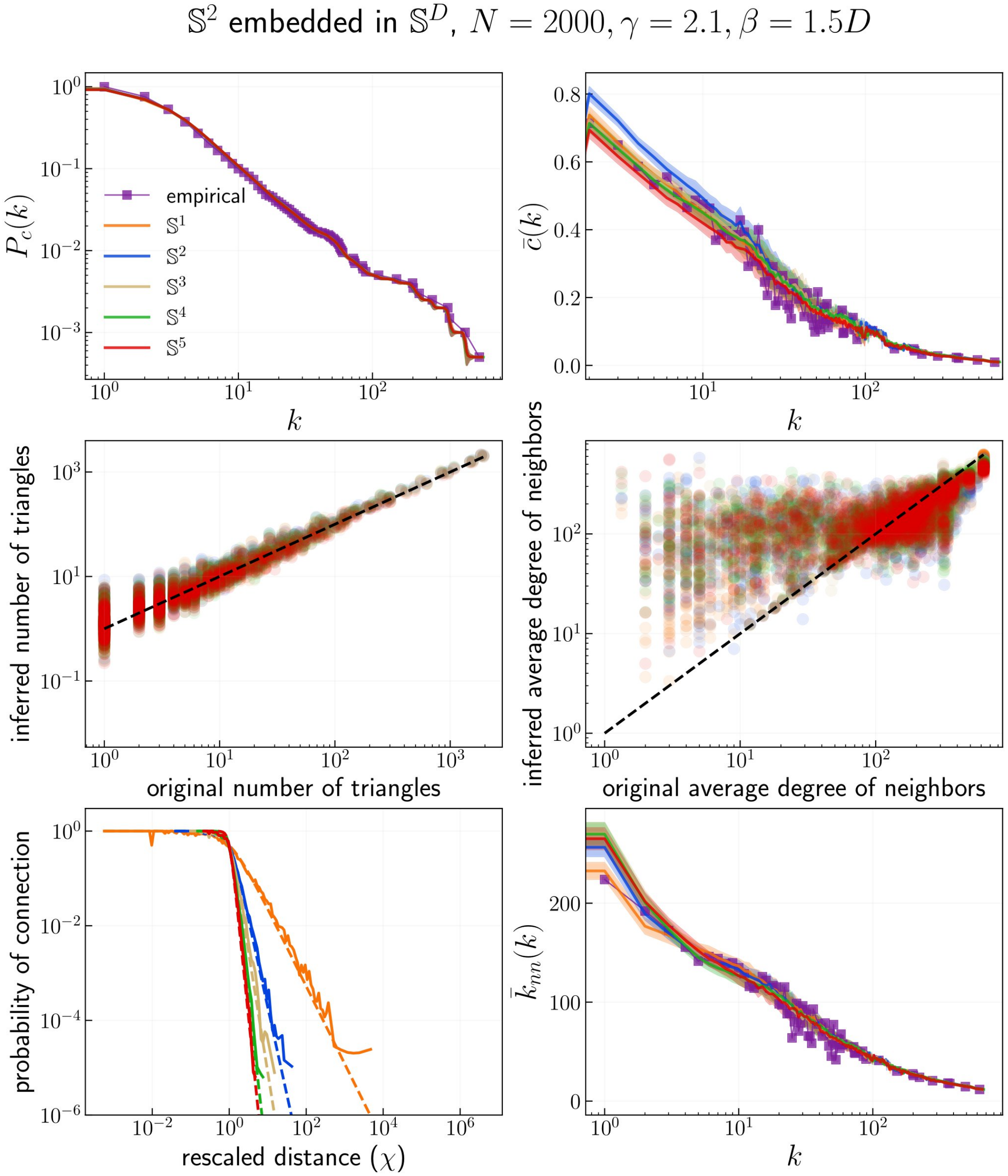

FIG. S6: Topological validation of the embeddings of the $\mathbb{S}^2$ model.

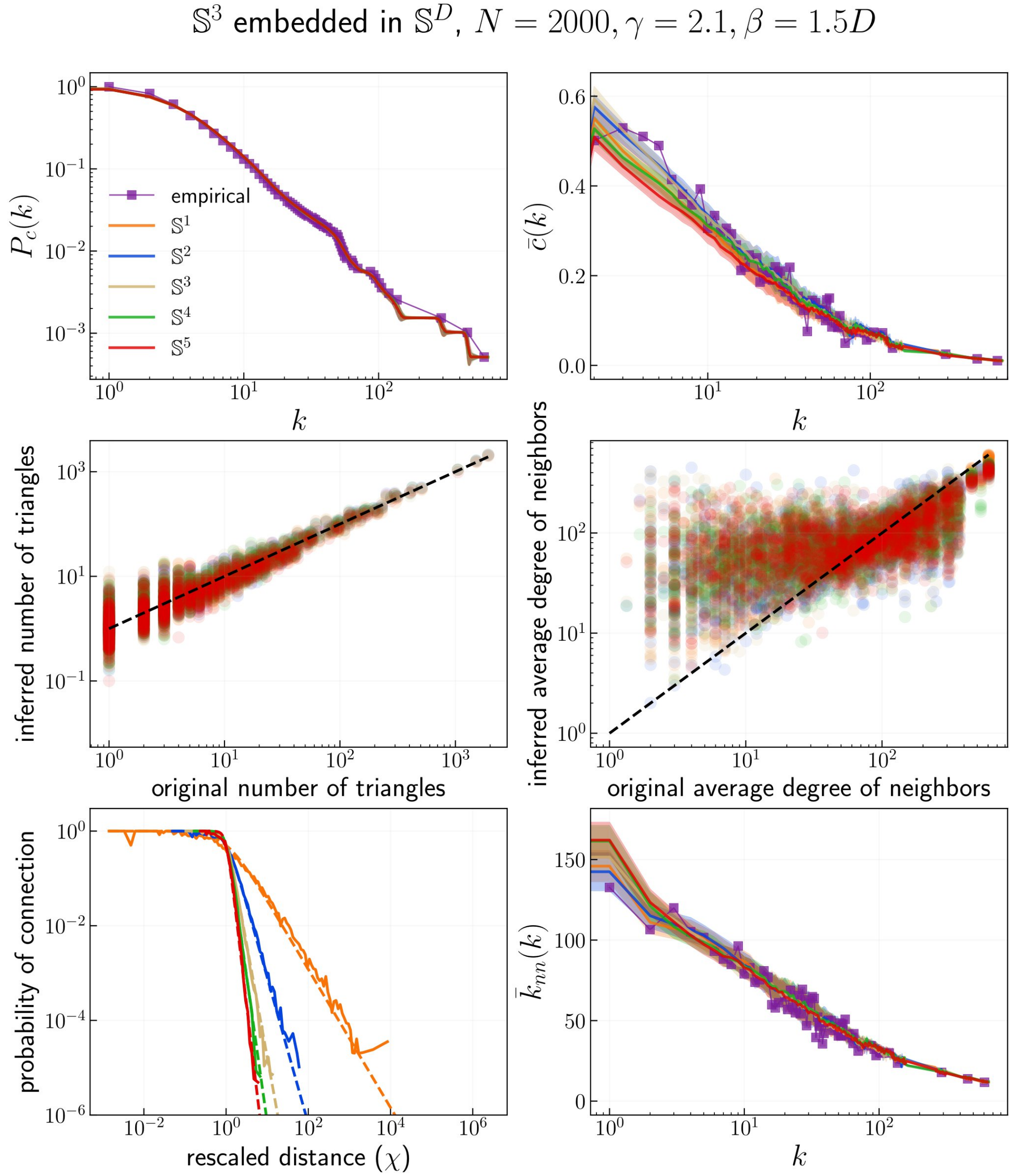

FIG. S7: Topological validation of the embeddings of the $\mathbb{S}^3$ model.

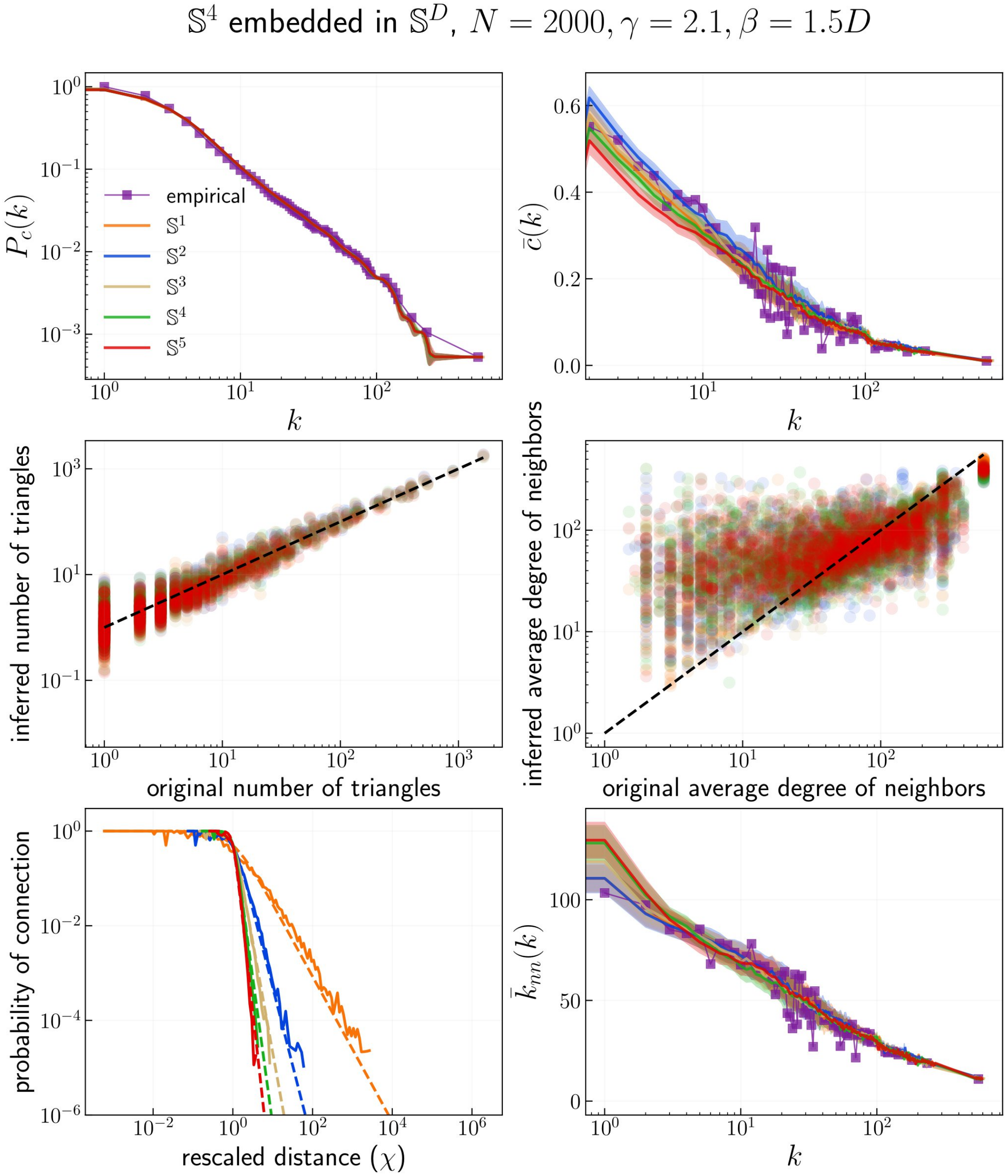

FIG. S8: Topological validation of the embeddings of the $\mathbb{S}^4$ model.

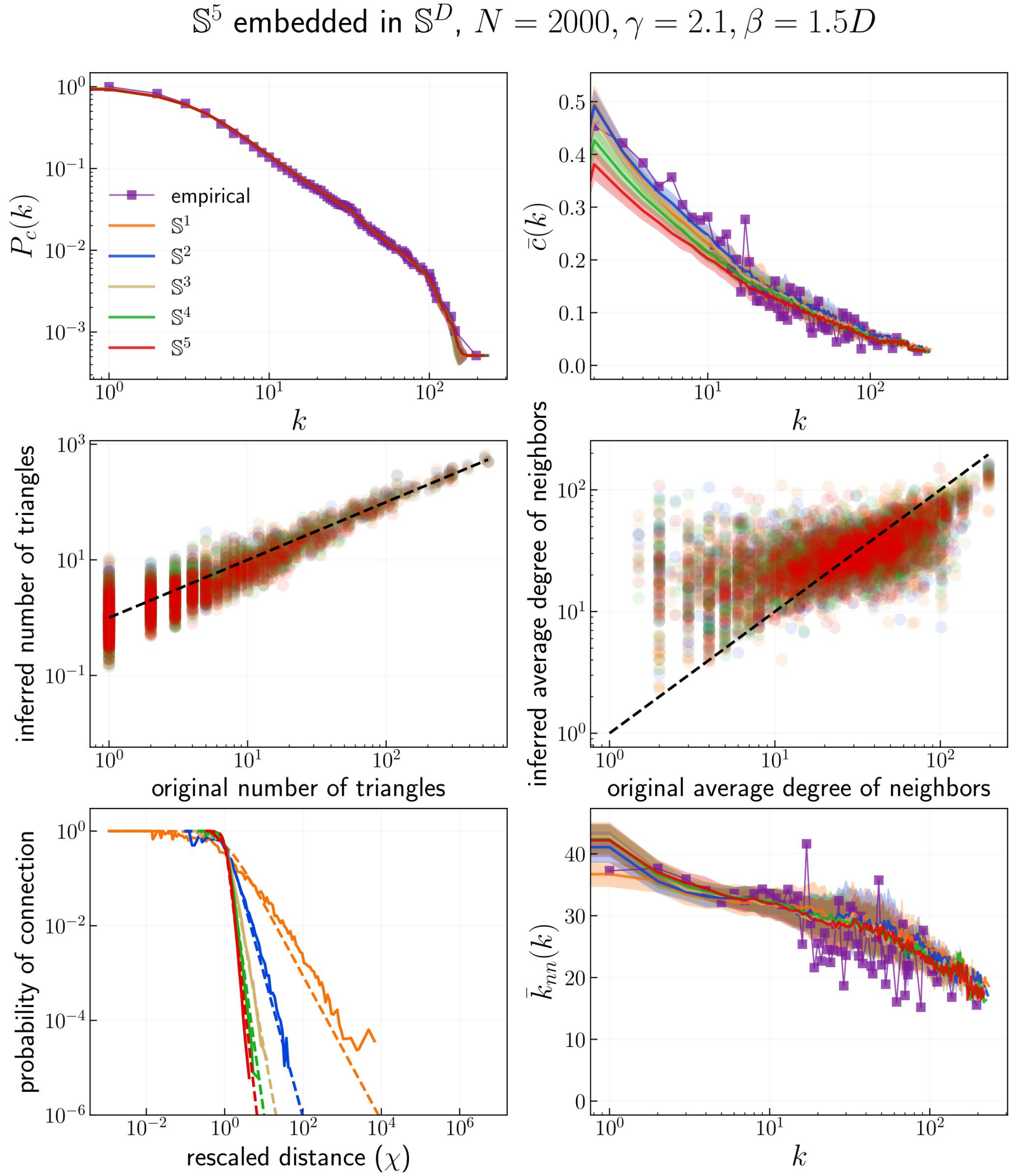

FIG. S9: Topological validation of the embeddings of the $\mathbb{S}^5$ model.

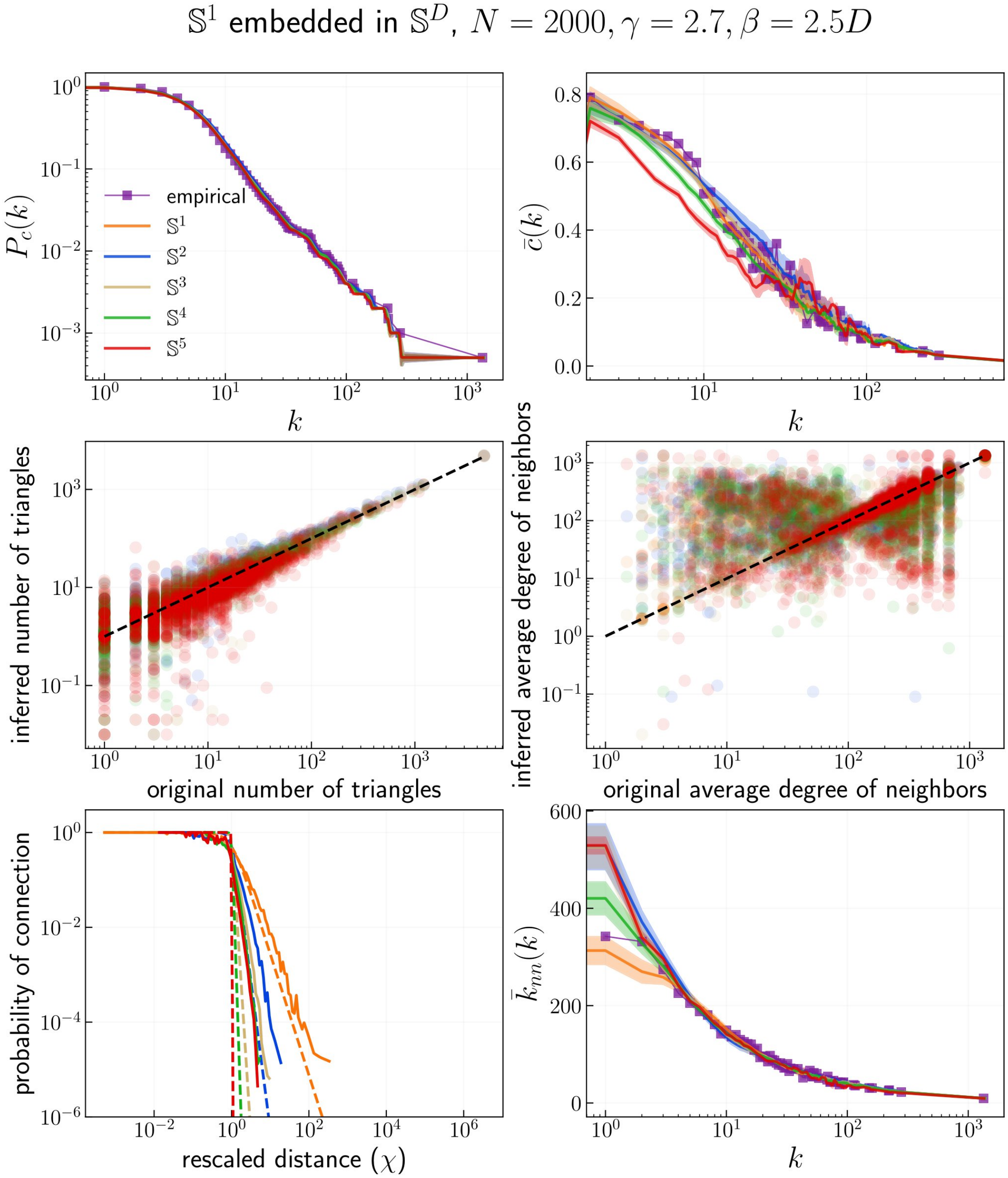

FIG. S10: Topological validation of the embeddings of the $\mathbb{S}^1$ model.

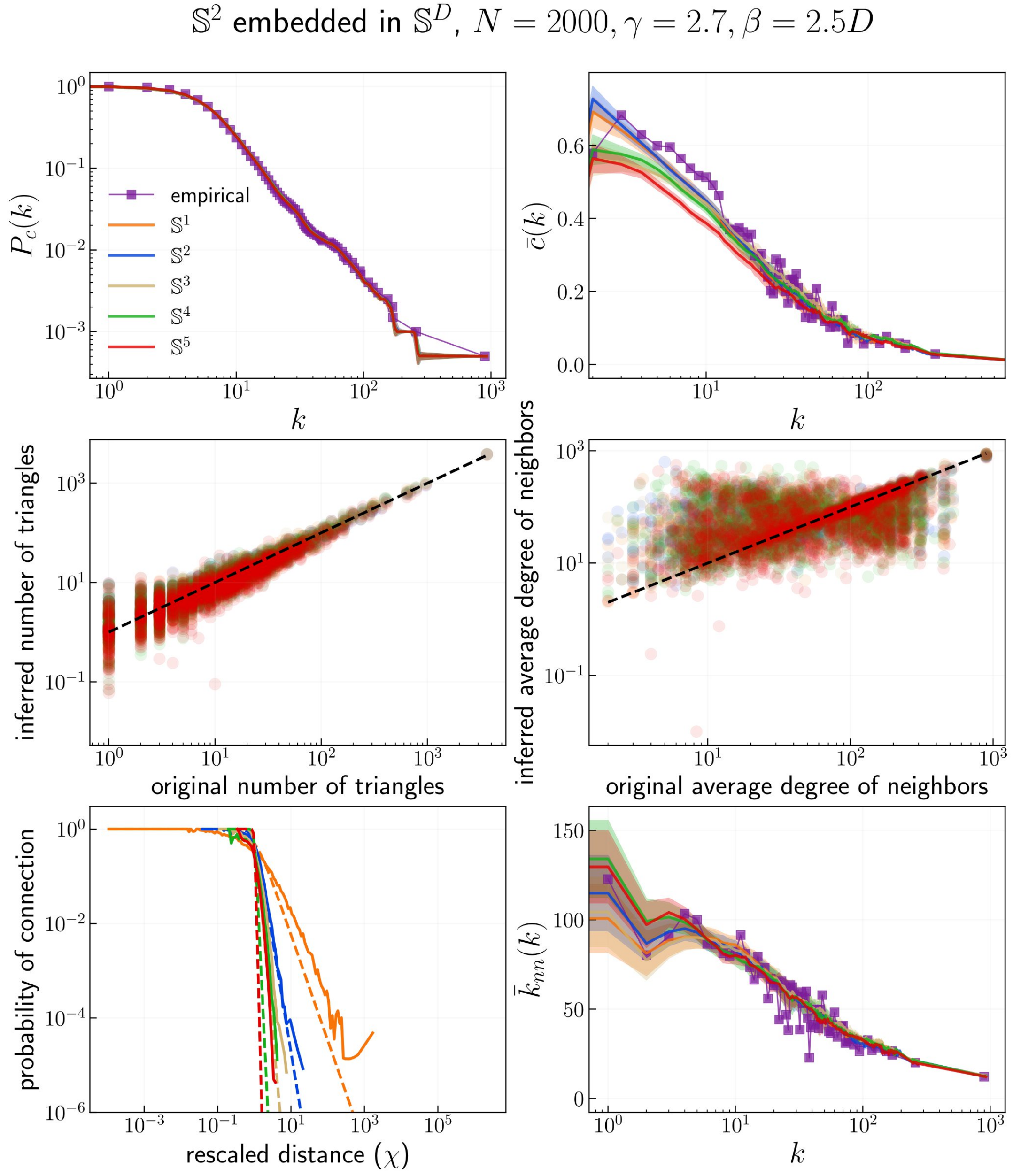

FIG. S11: Topological validation of the embeddings of the $\mathbb{S}^2$ model.

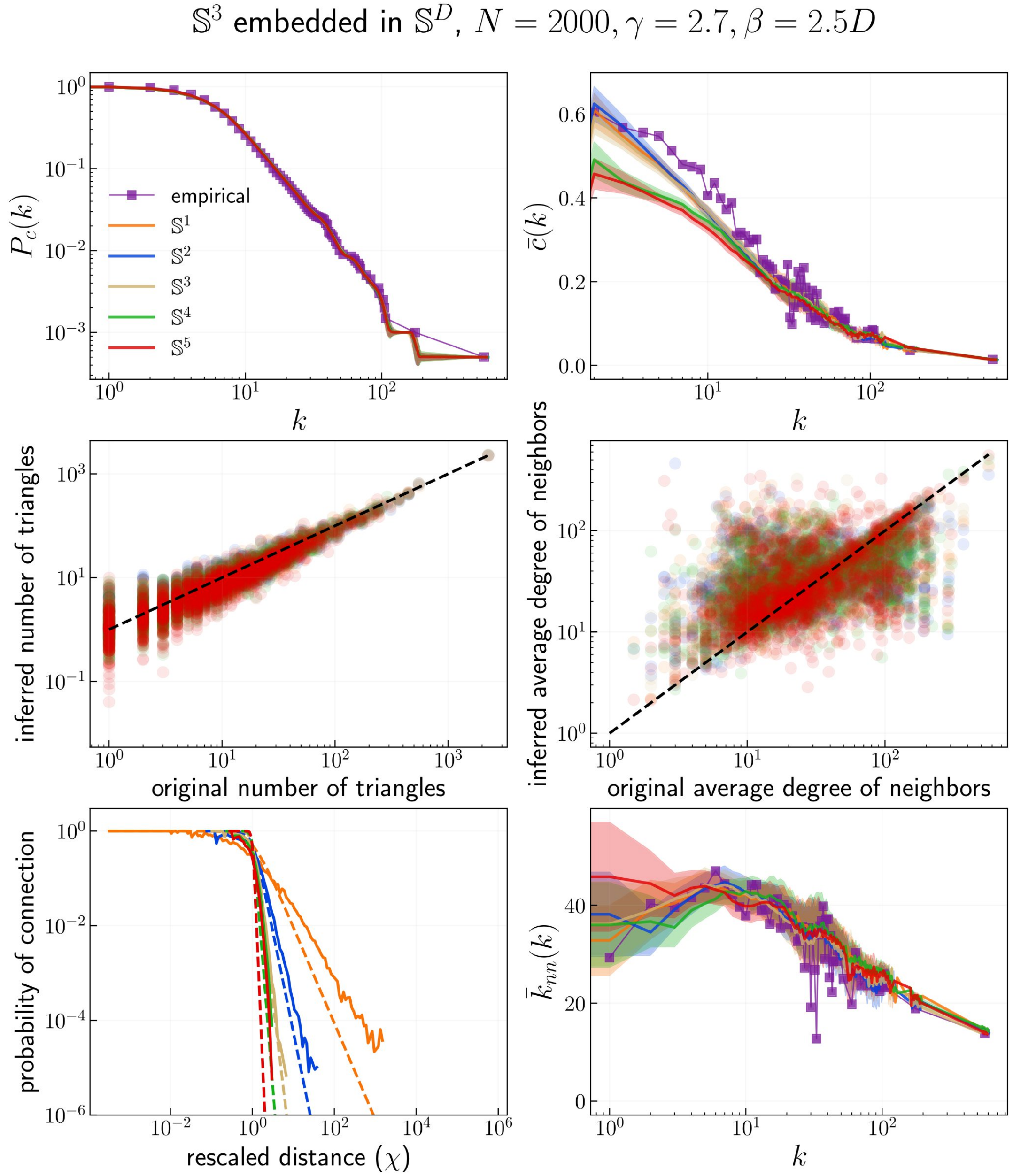

FIG. S12: Topological validation of the embeddings of the $\mathbb{S}^3$ model.

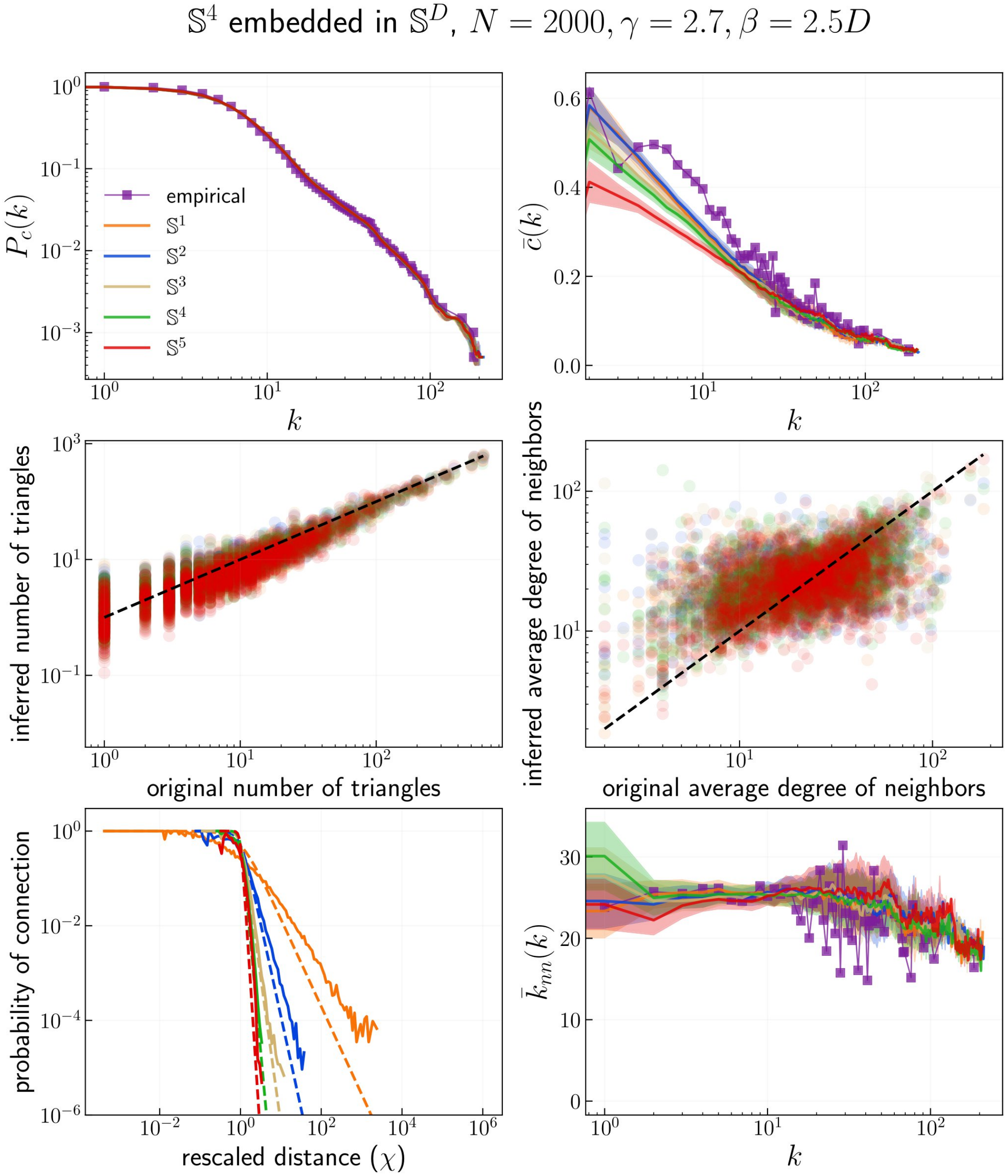

FIG. S13: Topological validation of the embeddings of the $\mathbb{S}^4$ model.

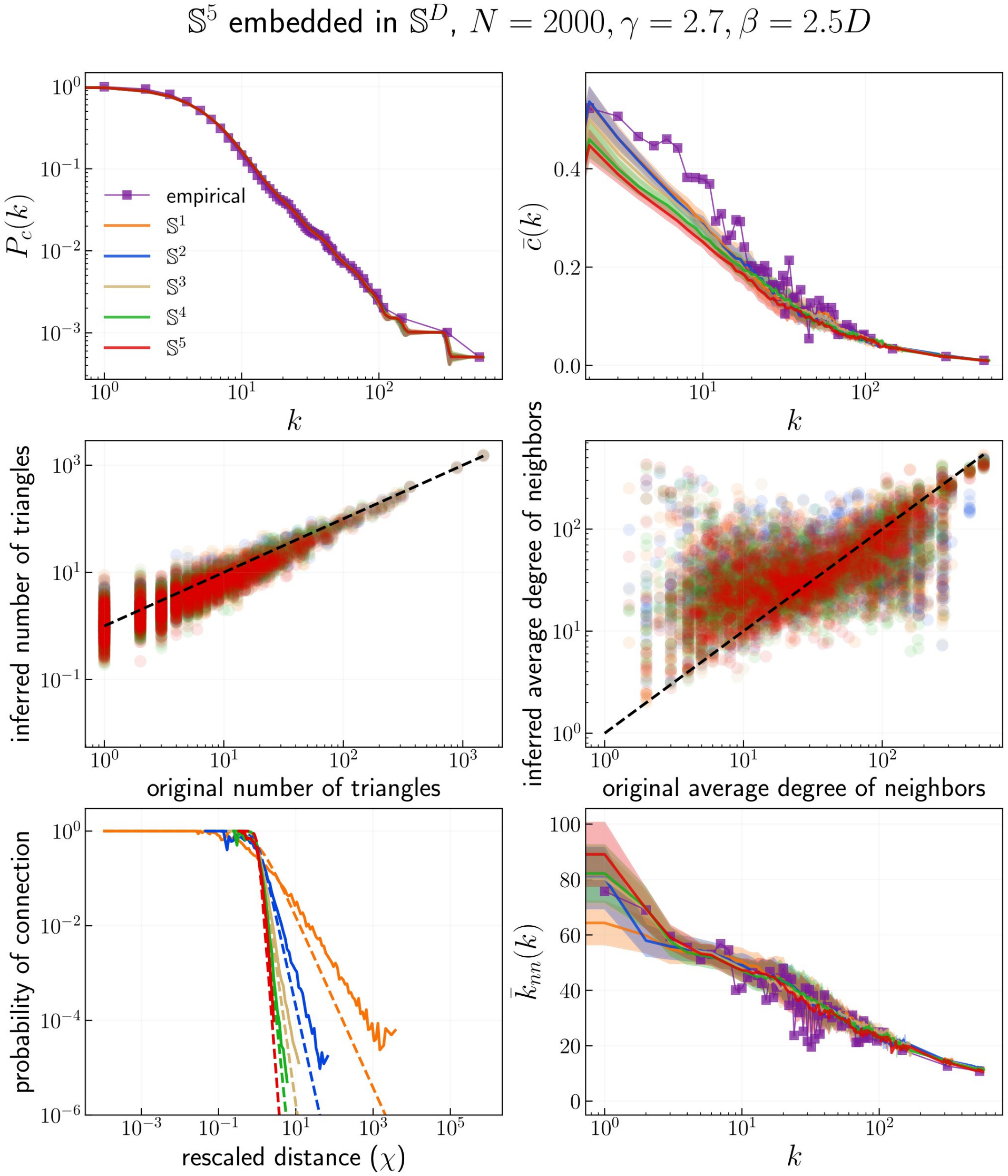

FIG. S14: Topological validation of the embeddings of the $\mathbb{S}^5$ model.

VIII. OTHER TOPOLOGICAL PROPERTIES OF NAVIGABILITY IN THE SYNTHETIC NETWORKS

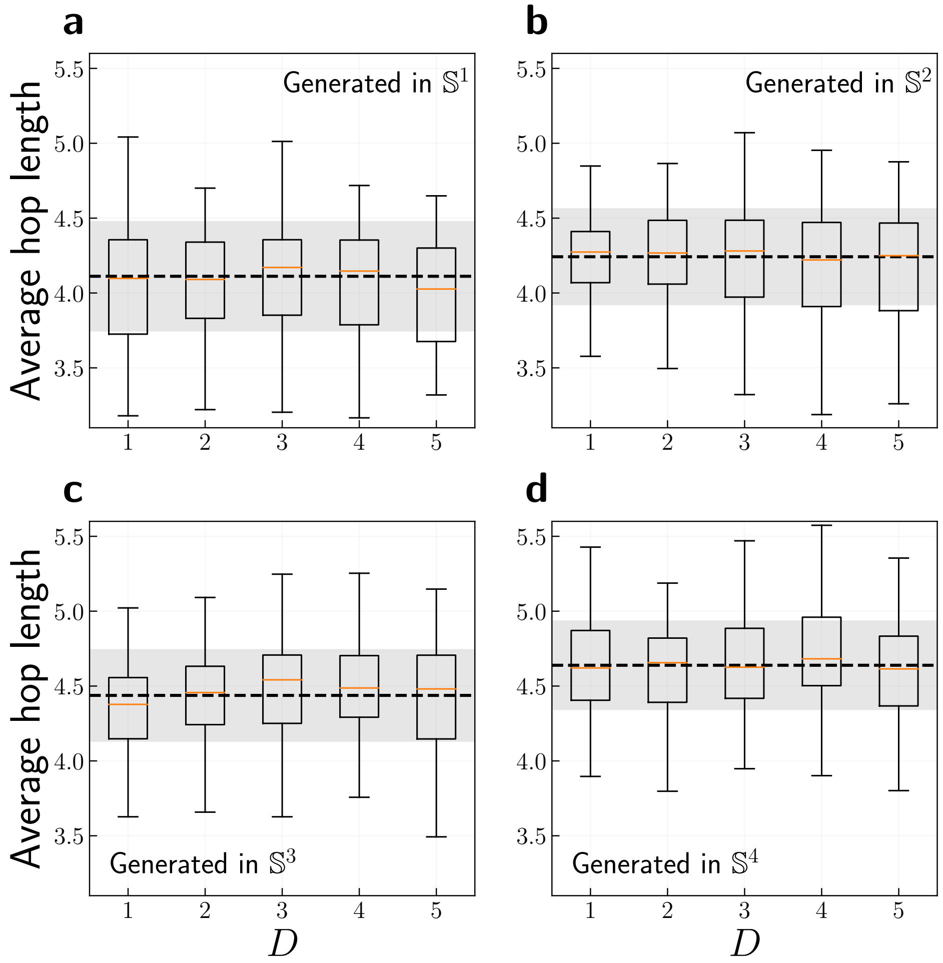

FIG. S15: Average hop length as a function of the embedding dimension for $\mathbb{S}^D$ synthetic networks generated in dimensions **(a)** $D = 1$, **(b)** $D = 2$, **(c)** $D = 3$ and **(d)** $D = 4$. The black lines show the maximum value of p_s for a given dimension and are computed from the generated networks, i.e., real coordinates of the synthetic networks. The box ranges from the first quartile to the third quartile. A vertical line goes through the box at the median. The whiskers go from each quartile to the minimum or maximum. The following parameters were used: $\beta = 2.5D, \gamma = 2.7, N = 2000$. Results are averaged over 100 realizations.

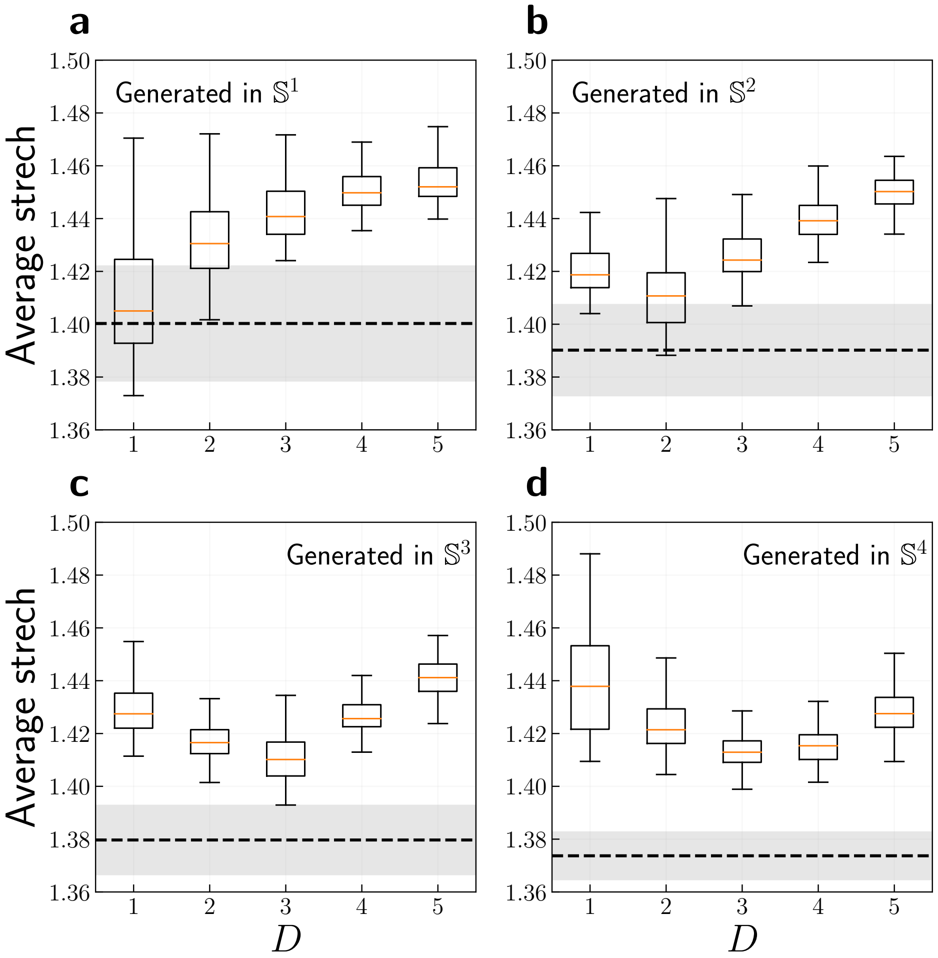

FIG. S16: Average stretch as a function of the embedding dimension for $\mathbb{S}^D$ synthetic networks generated in dimensions **(a)** $D = 1$, **(b)** $D = 2$, **(c)** $D = 3$ and **(d)** $D = 4$. See caption of Fig. S15 for more details.

IX. COMMUNITY CONCENTRATION OF SYNTHETIC NETWORKS WITH COMMUNITY STRUCTURE

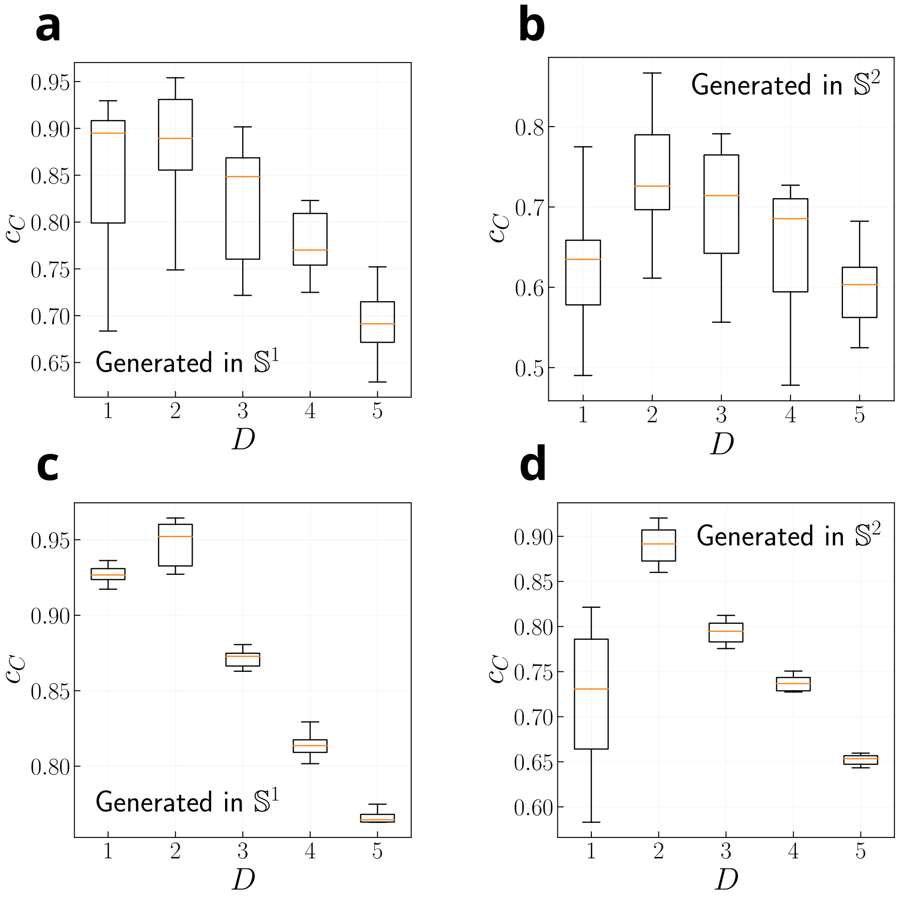

FIG. S17: The community concentration c_C for the $\mathbb{S}^1$ model with 4 communities **(a,c)** and $\mathbb{S}^2$ model with 6 communities **(b,d)** embedded in different dimensions. The following parameters were used to generate the networks: **(a,b)** $\beta = 2.5D, \gamma = 2.1, N = 2000$, **(c,d)** $\beta = 1.5D, \gamma = 3.5, N = 2000$. Results are averaged over 10 realizations.

A. Community overlap

To measure the overlap of nodes in $\mathbb{S}^1$ and $\mathbb{S}^2$ models with community structure, we define a simple measure that computes the distance between a given node and all the centers. If a node is not closer to its center, we count it as an overlap. The detailed algorithm is shown as follows.

1. For each node i with label l.
2. Compute $\Delta\theta_{il}$, which is a distance from node i to its center l.
3. Compute all angular distances to the rest of the centers.
4. If a node i is closer to any other centers then l, mark this node as being the overlap.
5. Repeat for every node and compute the average.

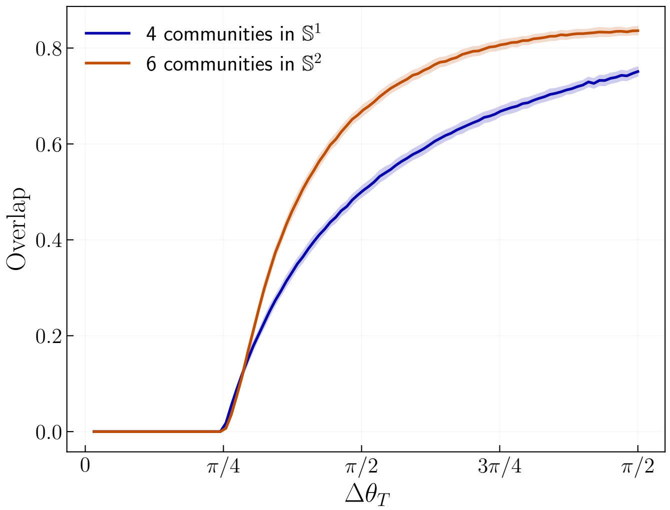

FIG. S18: Overlap of communities as the function of $\Delta\theta_T$ in the synthetic networks. The results are averaged over 100 realizations.

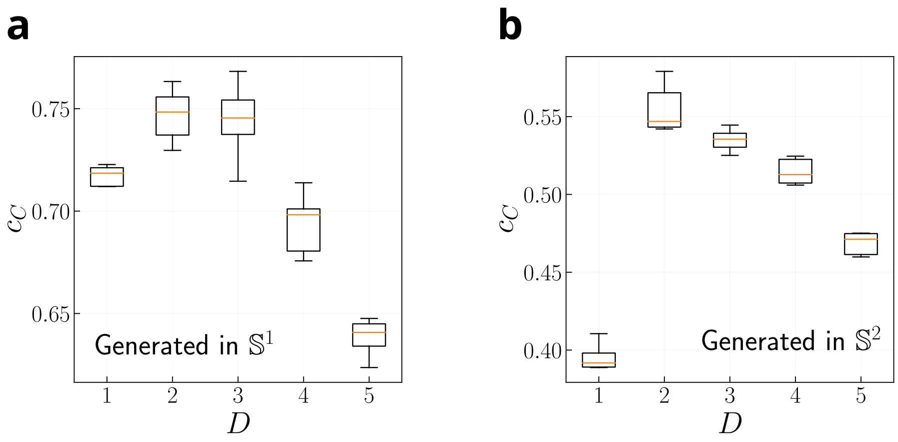

FIG. S19: The community concentration c_C for the **(a)** $\mathbb{S}^1$ model with 4 communities and **(b)** $\mathbb{S}^2$ model with 6 communities embedded in different dimensions when communities were overlapping, i.e., with $\Delta\theta = 1.0$. The following parameters were used to generate the networks: $\beta = 1.5D, \gamma = 2.7, N = 2000$. Results are averaged over 10 realizations.

X. TOPOLOGICAL PROPERTIES OF SYNTHETIC NETWORKS WITH COMMUNITY STRUCTURE

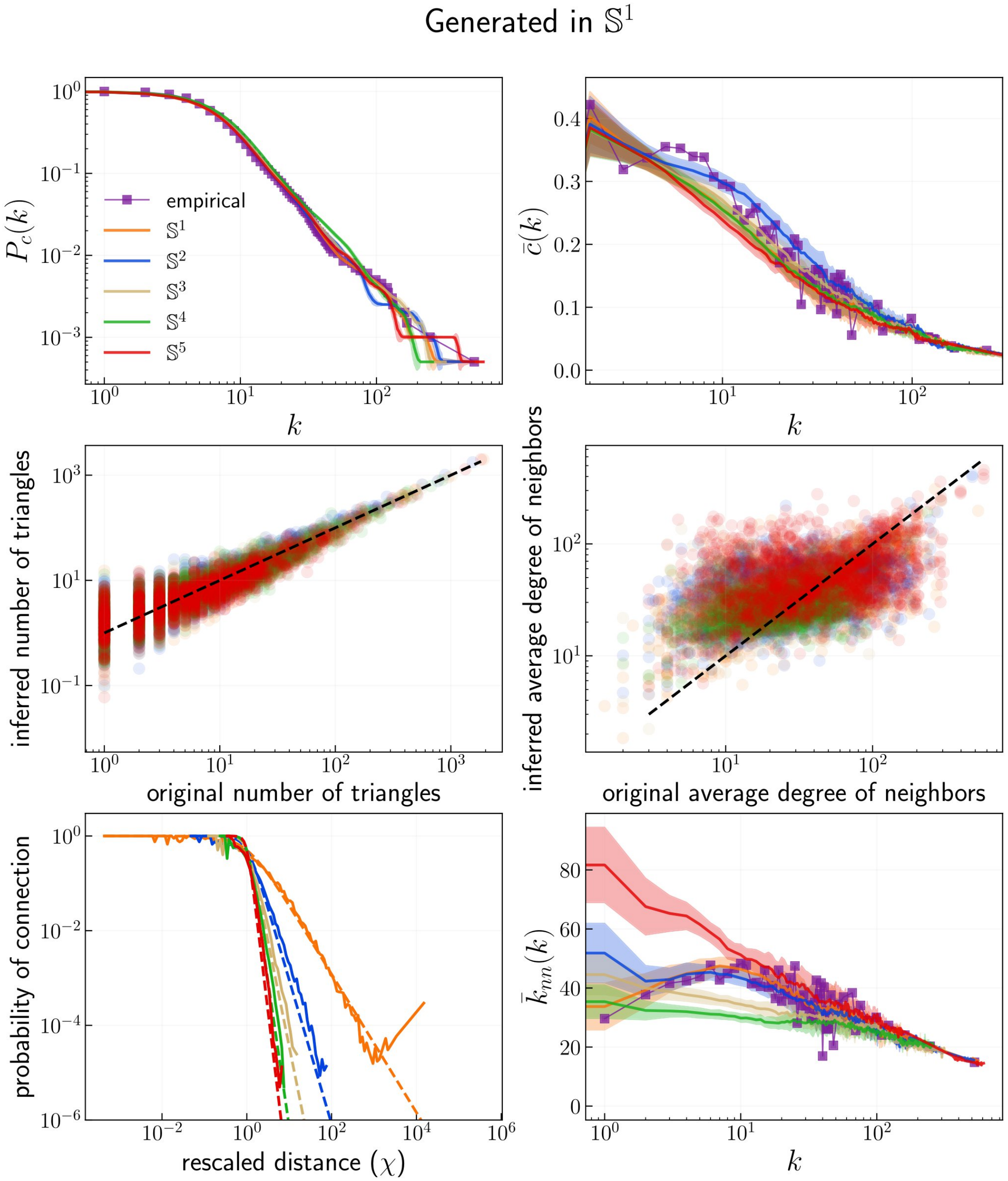

FIG. S20: Topological validation of the embeddings of the $\mathbb{S}^1$ model with community structure embedded in different dimensions.

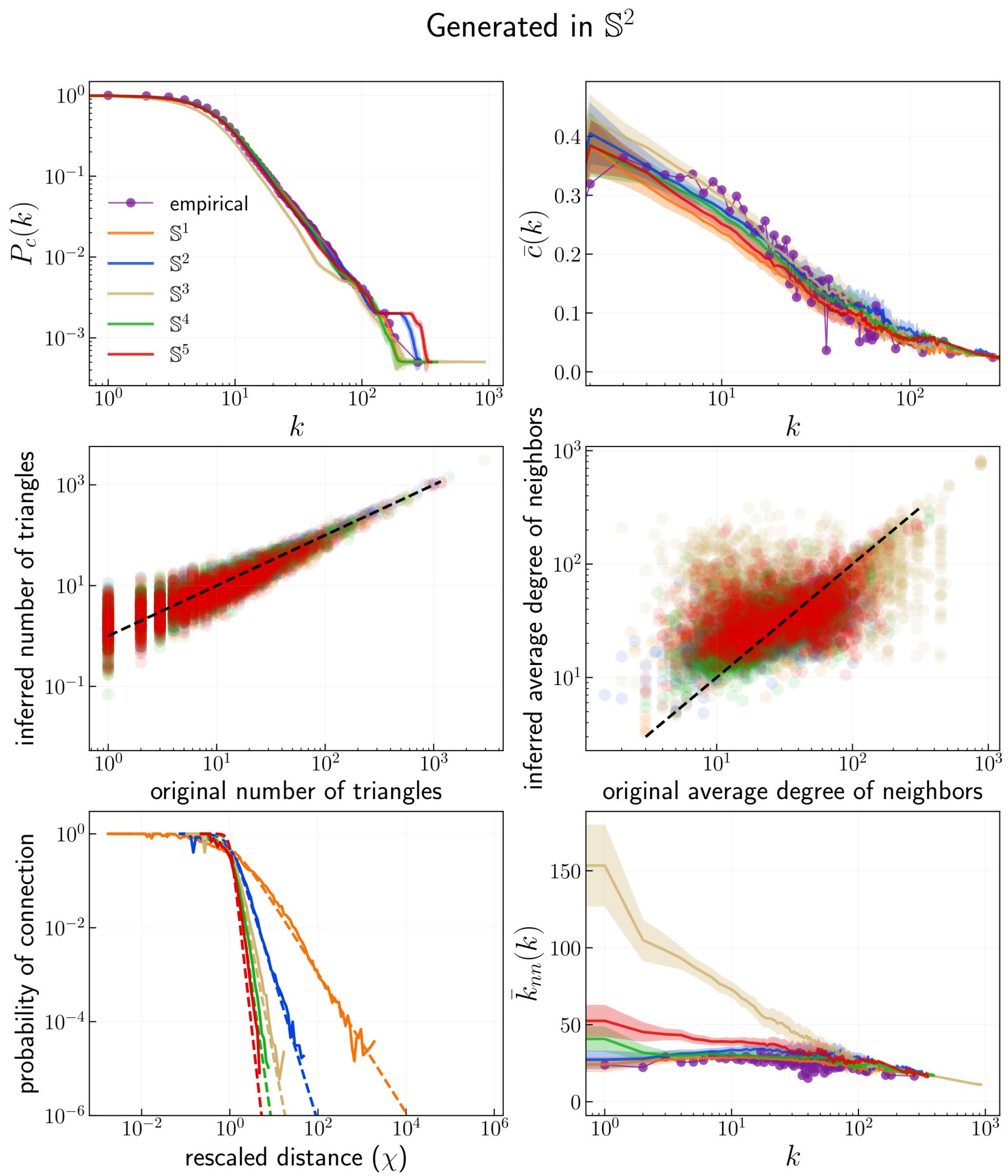

FIG. S21: Topological validation of the embeddings of the $\mathbb{S}^2$ model with community structure embedded in different dimensions.

XI. PROPERTIES OF REAL NETWORKS

Network	N	$\langle k\rangle$	$\bar{c}$	N_C	β_1	β_2	β_3	β_4	β_5	μ_1	μ_2	μ_3	μ_4	μ_5
Add-health	1996	8.54	0.15	6	1.29	2.62	3.90	5.30	6.44	0.0156	0.0105	0.0077	0.007	0.0059
FAO-apples	152	15.99	0.56	6	1.02	3.98	5.19	5.53	5.64	0.0006	0.0126	0.008	0.0043	0.002
C. elegans	559	16.1	0.32	5	1.52	3.16	4.69	6.23	8.05	0.0132	0.0091	0.0067	0.0056	0.0056
OpenFlights	2905	10.77	0.59	6	1.85	3.82	5.97	7.99	10.65	0.0272	0.0179	0.014	0.012	0.0119
Foxglove	2916	11.21	0.43	8	2.31	5.11	10.23	22.44	$100^\dagger$	0.0320	0.0218	0.0184	0.0172	0.0169
Polbooks	105	8.4	0.49	3	2.21	4.75	8.03	12.12	18.83	0.0415	0.0278	0.0223	0.02	0.0201

TABLE S1: Properties of selected real networks. The N_C indicates the number of ground-truth communities. The β_i and μ_i values are presented for the i's dimension of the embeddings. The † indicates that β could not be inferred in that dimension.

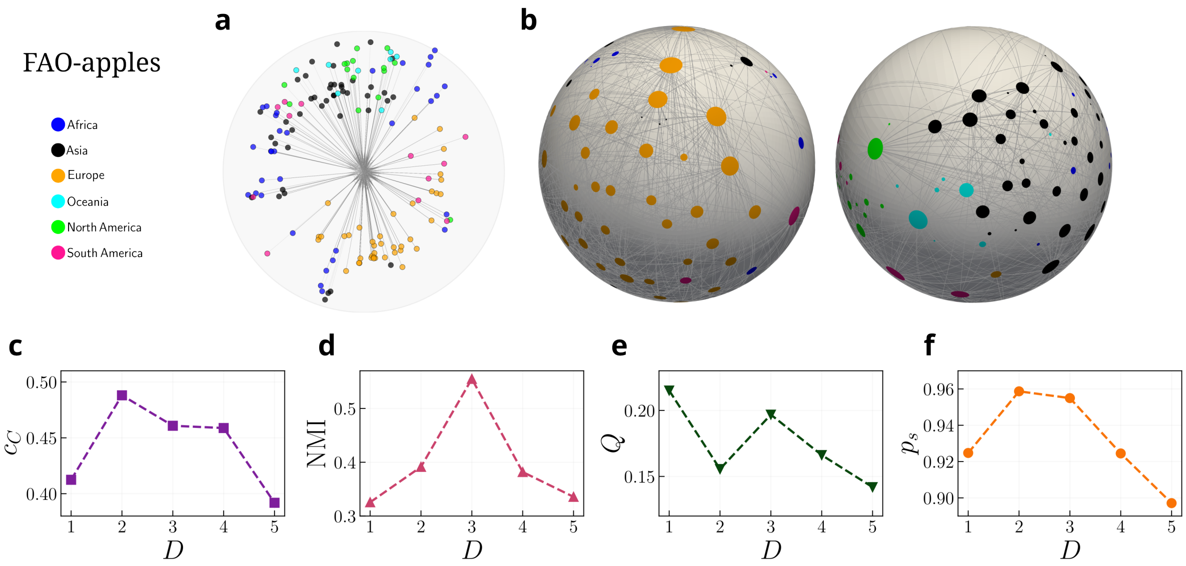

FIG. S22: **Case study: the FAO-apples dataset**. Top panels show the embeddings in (**a**) $D = 1$ and (**b**) two perspectives of the $D = 2$ similarity space of D-Mercator embeddings of the network. The size of a node is proportional to its expected degree, and its color indicates the community it belongs to. For the sake of clarity, only the connections with probability $p_{ij} > 0.5$ given by Eq. 1 from main text are shown. Bottom panels show the performance of (**c**) community concentration (c_C), (**d**) community detection (NMI), (**e**) modularity (Q), and (**f**) the success rate of GR (p_s).

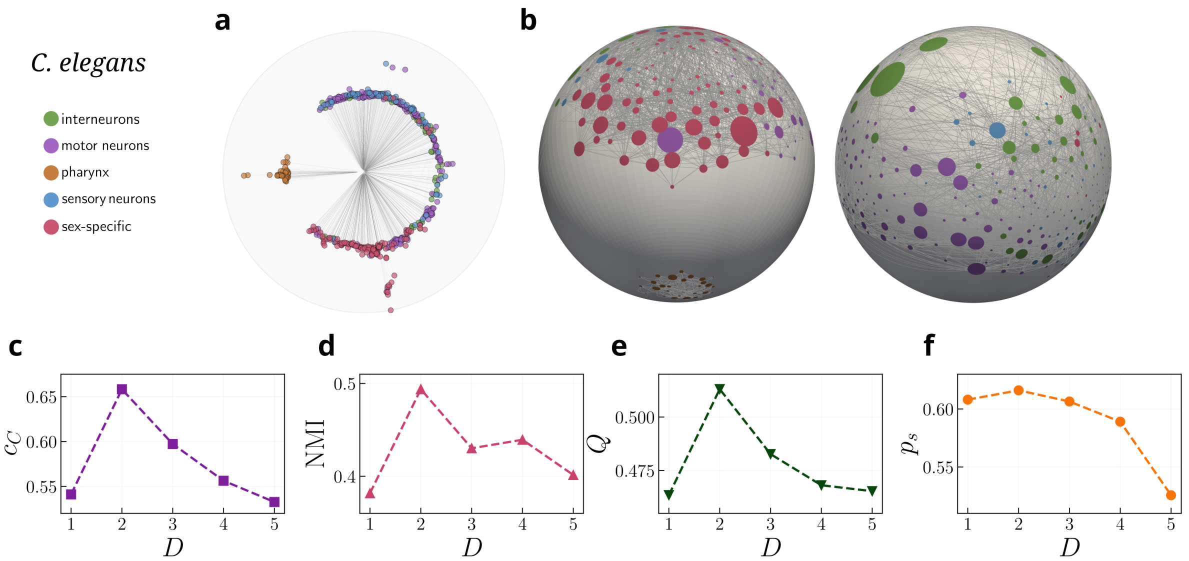

FIG. S23: **Case study: the *C. elegans* dataset**. Top panels show the embeddings in (**a**) $D = 1$ and (**b**) two perspectives of the $D = 2$ similarity space of D-Mercator embeddings of the network. The size of a node is proportional to its expected degree, and its color indicates the community it belongs to. For the sake of clarity, only the connections with probability $p_{ij} > 0.5$ given by Eq. 1 from main text are shown. Bottom panels show the performance of (**c**) community concentration (c_C), (**d**) community detection (NMI), (**e**) modularity (Q), and (**f**) the success rate of GR (p_s).

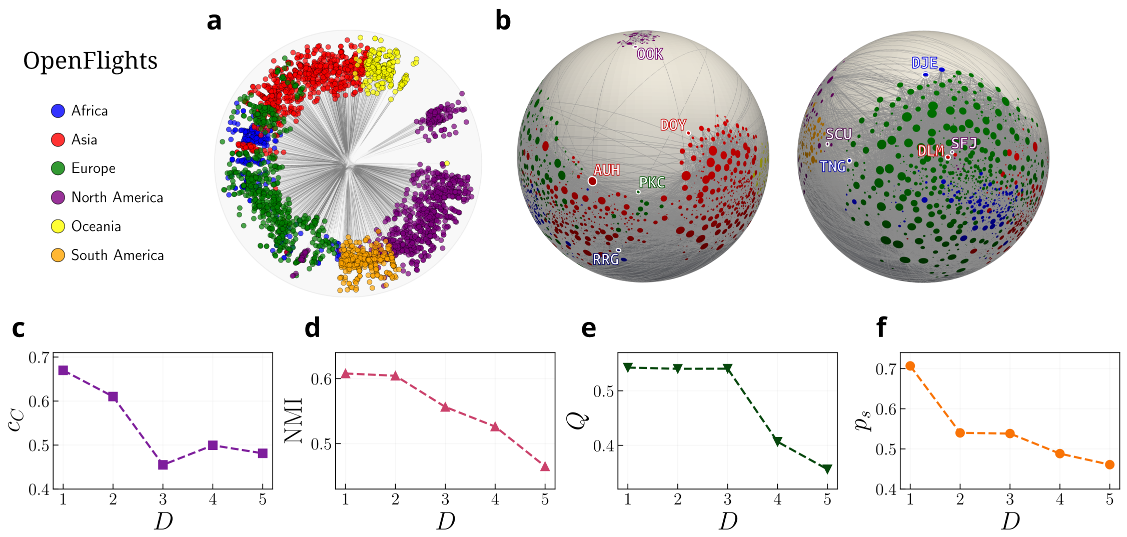

FIG. S24: **Case study: the Openflights dataset**. Top panels show the embeddings in (**a**) $D = 1$ and (**b**) two perspectives of the $D = 2$ similarity space of D-Mercator embeddings of the network. **AUH** – Abu Dhabi International Airport (United Arab Emirates), **RRG** – Plaine Corail Airport (Mauritius), **PKC** – Yelizovo Airport (Russian Federation), **OOK** – Toksook Bay Airport (United States), **DOY** – Dongying Shengli Airport (China), **SCU** – Antonio Maceo International Airport (Cuba), **TNG** – Ibn Batouta International Airport (Morocco), **DJE** – Zarzis Airport (Tunisia), **DLM** – Dalaman Airport (Turkey), **SFJ** – Kangerlussuaq Airport (Greenland). The size of a node is proportional to its expected degree, and its color indicates the community it belongs to. For the sake of clarity, only the connections with probability $p_{ij} > 0.5$ given by Eq. 1 from main text are shown. Bottom panels show the performance of (**c**) community concentration (c_C), (**d**) community detection (NMI), (**e**) modularity (Q), and (**f**) the success rate of GR (p_s).

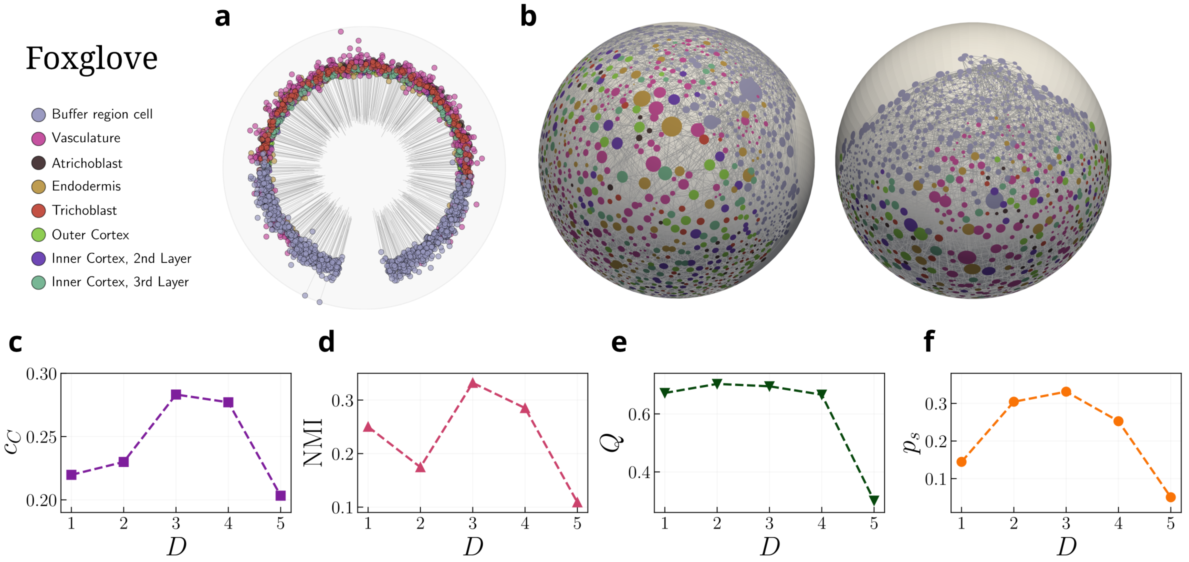

FIG. S25: **Case study: the Foxglove dataset**. Top panels show the embeddings in (**a**) $D = 1$ and (**b**) two perspectives of the $D = 2$ similarity space of D-Mercator embeddings of the network. The size of a node is proportional to its expected degree, and its color indicates the community it belongs to. For the sake of clarity, only the connections with probability $p_{ij} > 0.5$ given by Eq. 1 from main text are shown. Bottom panels show the performance of (**c**) community concentration (c_C), (**d**) community detection (NMI), (**e**) modularity (Q), and (**f**) the success rate of GR (p_s).

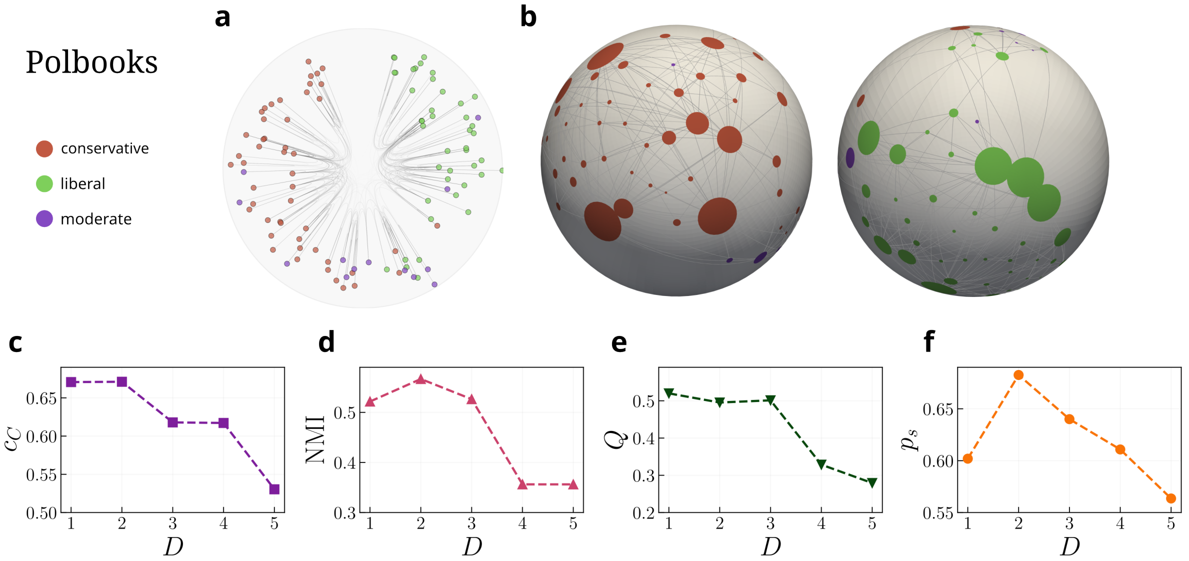

FIG. S26: **Case study: the Polbooks dataset**. Top panels show the embeddings in (**a**) $D = 1$ and (**b**) two perspectives of the $D = 2$ similarity space of D-Mercator embeddings of the network. The size of a node is proportional to its expected degree, and its color indicates the community it belongs to. For the sake of clarity, only the connections with probability $p_{ij} > 0.5$ given by Eq. 1 from main text are shown. Bottom panels show the performance of (**c**) community concentration (c_C), (**d**) community detection (NMI), (**e**) modularity (Q), and (**f**) the success rate of GR (p_s).

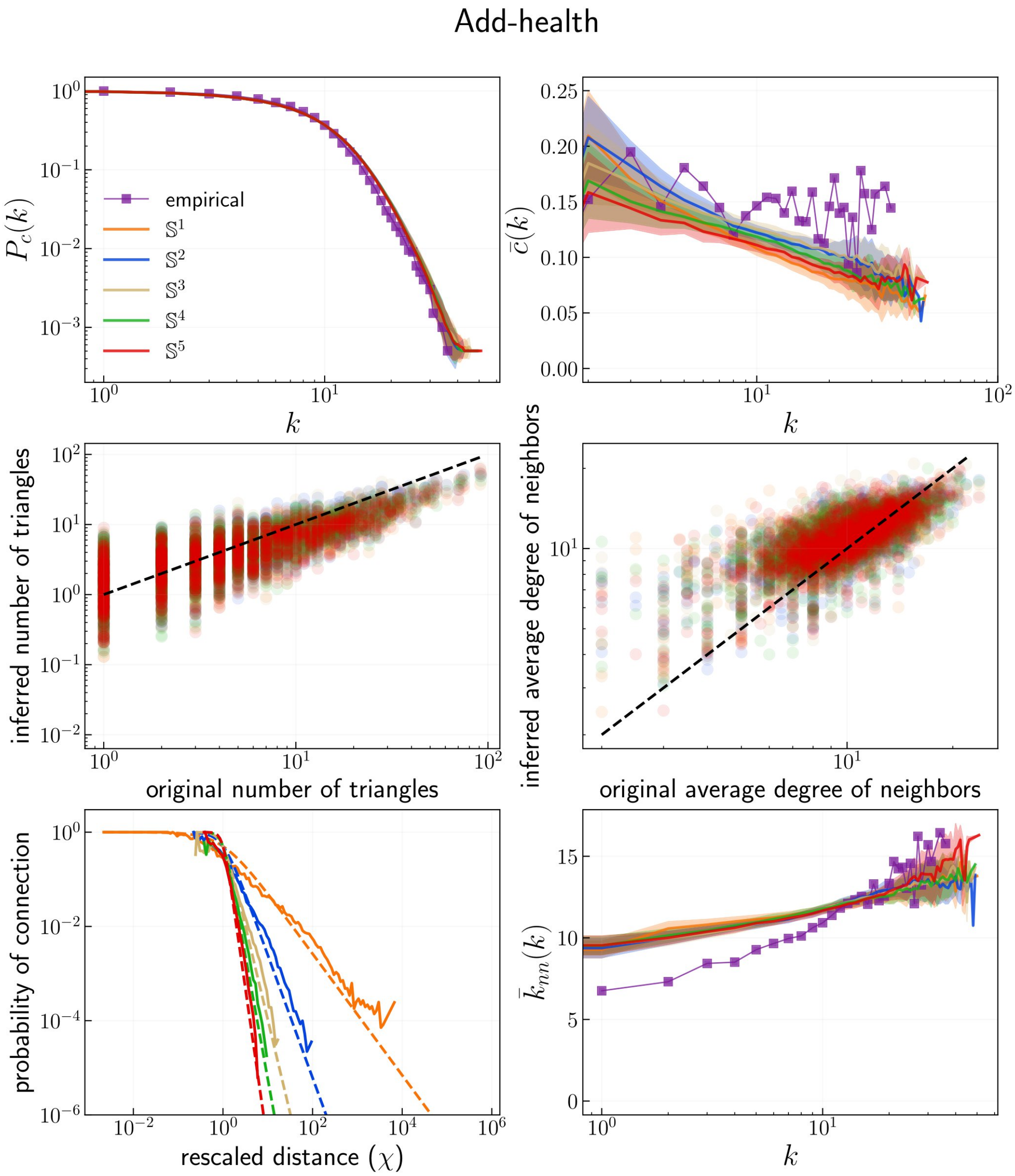

FIG. S27: Topological validation of the embeddings of the Add-health network. The first row shows the complementary cumulative degree distribution and the clustering spectrum $\bar{c}(k)$. Symbols correspond to the value of these quantities in the original network, whereas the lines indicate an estimate of their expected values in the ensemble of random networks in a given dimension inferred by D-Mercator. This ensemble was sampled by generating 100 synthetic networks with the $\mathbb{S}^D$ model and the inferred parameters and positions by D-Mercator. The error bars show the 2σ confidence interval around the expected value. The second row shows scattered plots of the sum of the degrees of their neighbors and the number of triangles to which they participate. The plots show the estimated values of these two measures in the same ensemble of random networks considered above versus the corresponding values in the original network. The last row depicts the comparison of the expected connection probability based on the inferred value of β (expected) and the actual connection probability computed with the inferred hidden variables. Whereas on the right, the plot shows the average nearest neighbors degree $\bar{k}_{nn}(k)$.

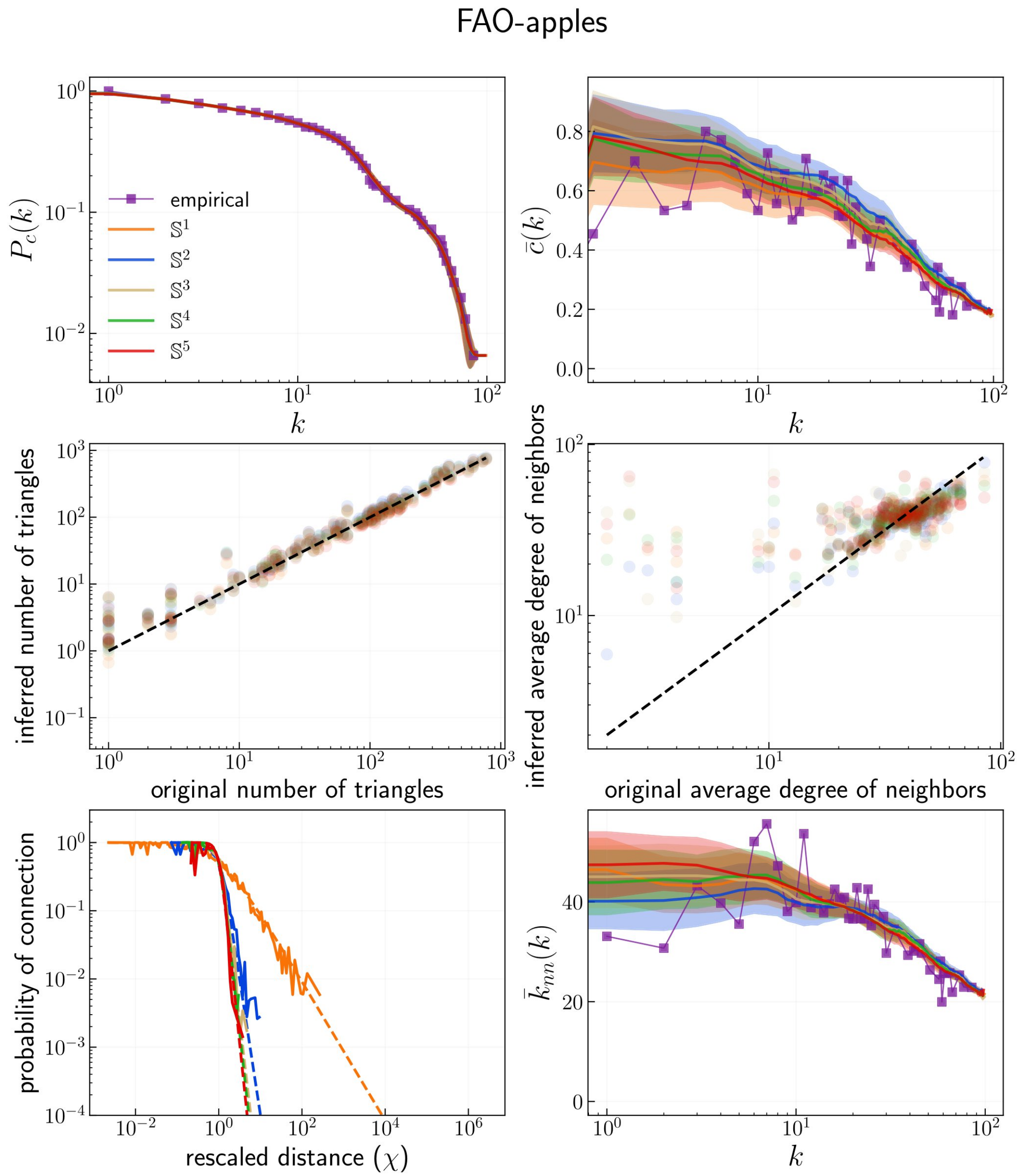

FIG. S28: Topological validation of the embeddings of the FAO-apples network. See caption in Fig. S27 for more details.

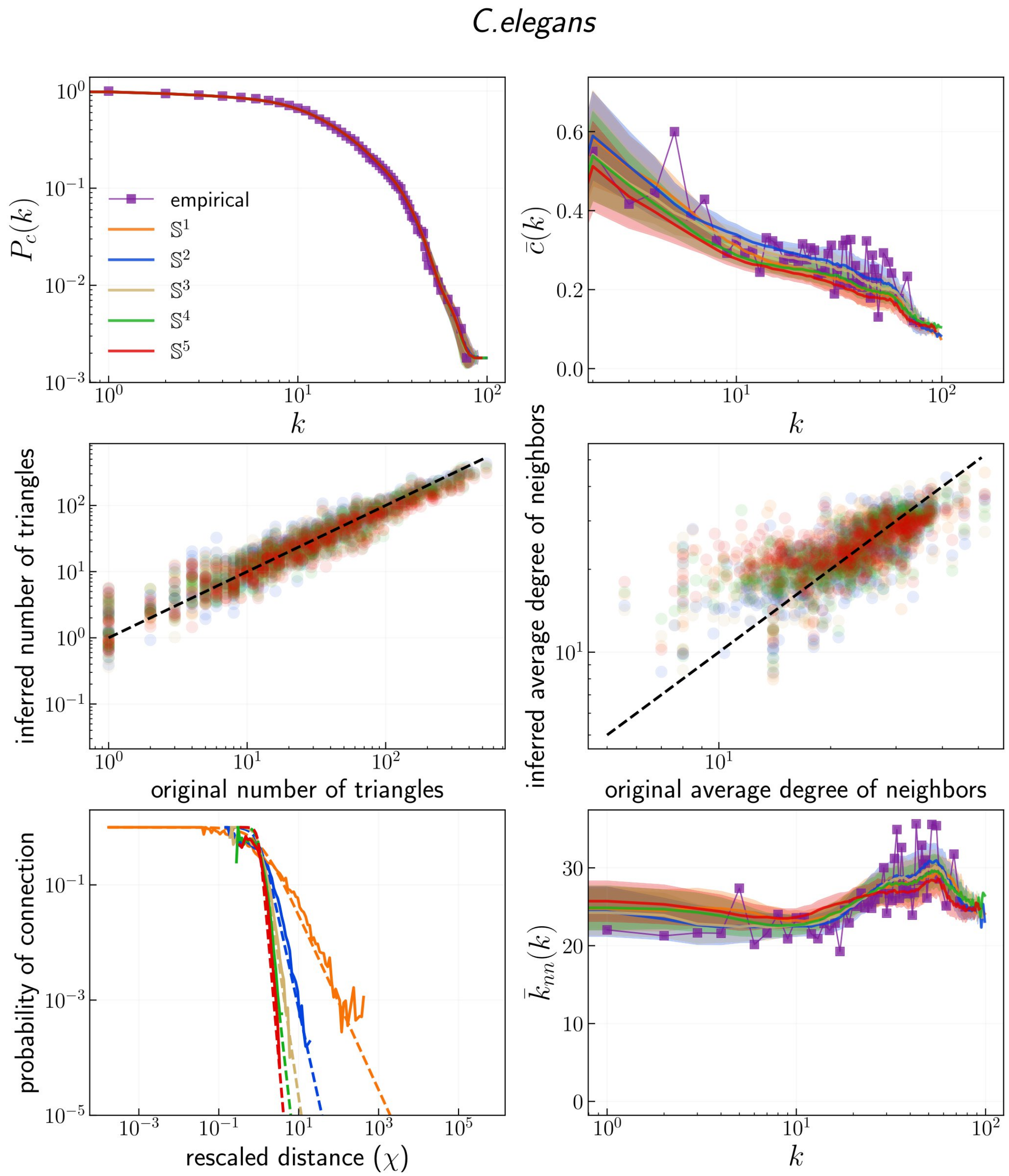

FIG. S29: Topological validation of the embeddings of the *C. elegans* network. See caption in Fig. S27 for more details.

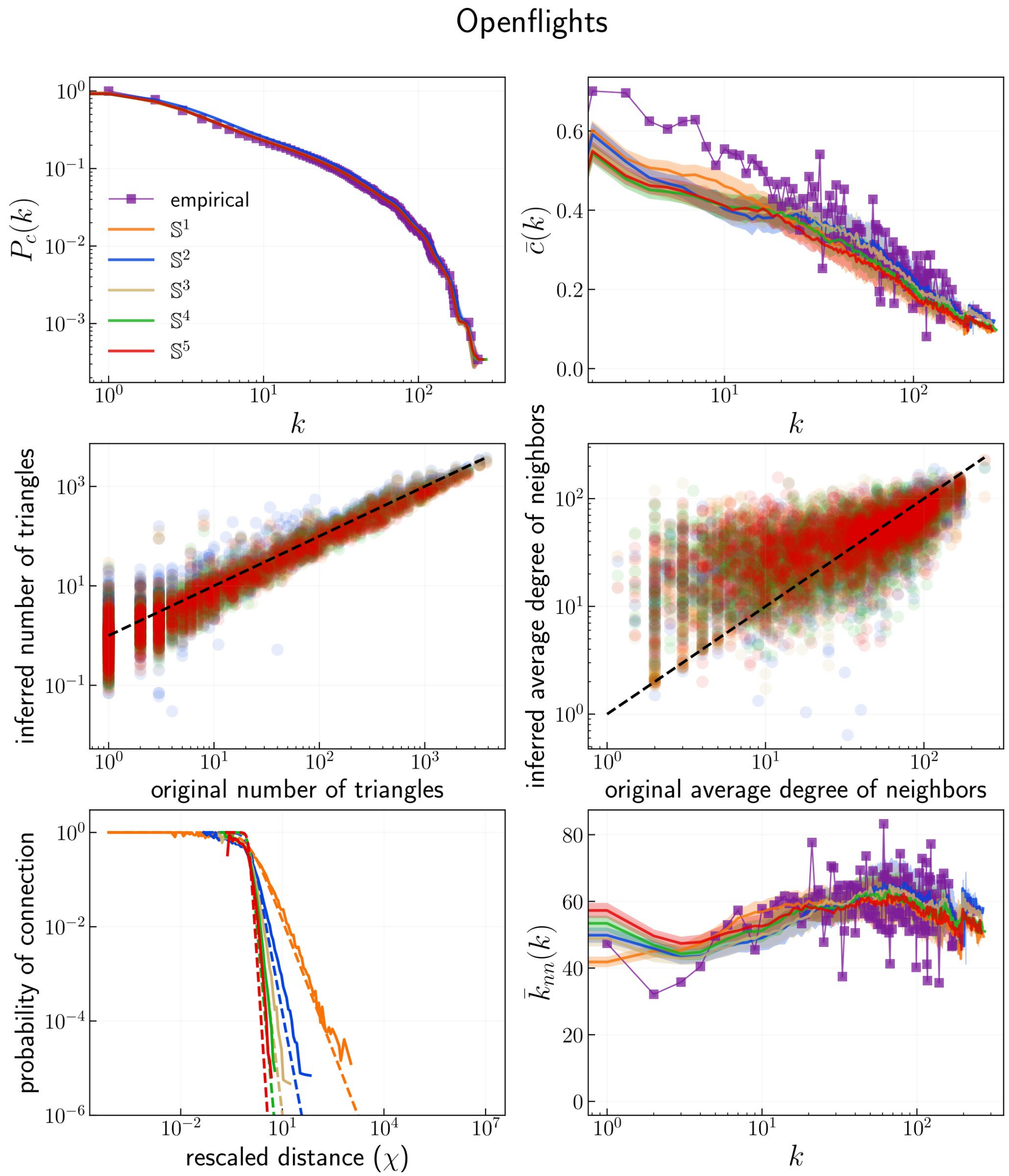

FIG. S30: Topological validation of the embeddings of the OpenFlights network. See caption in Fig. S27 for more details.

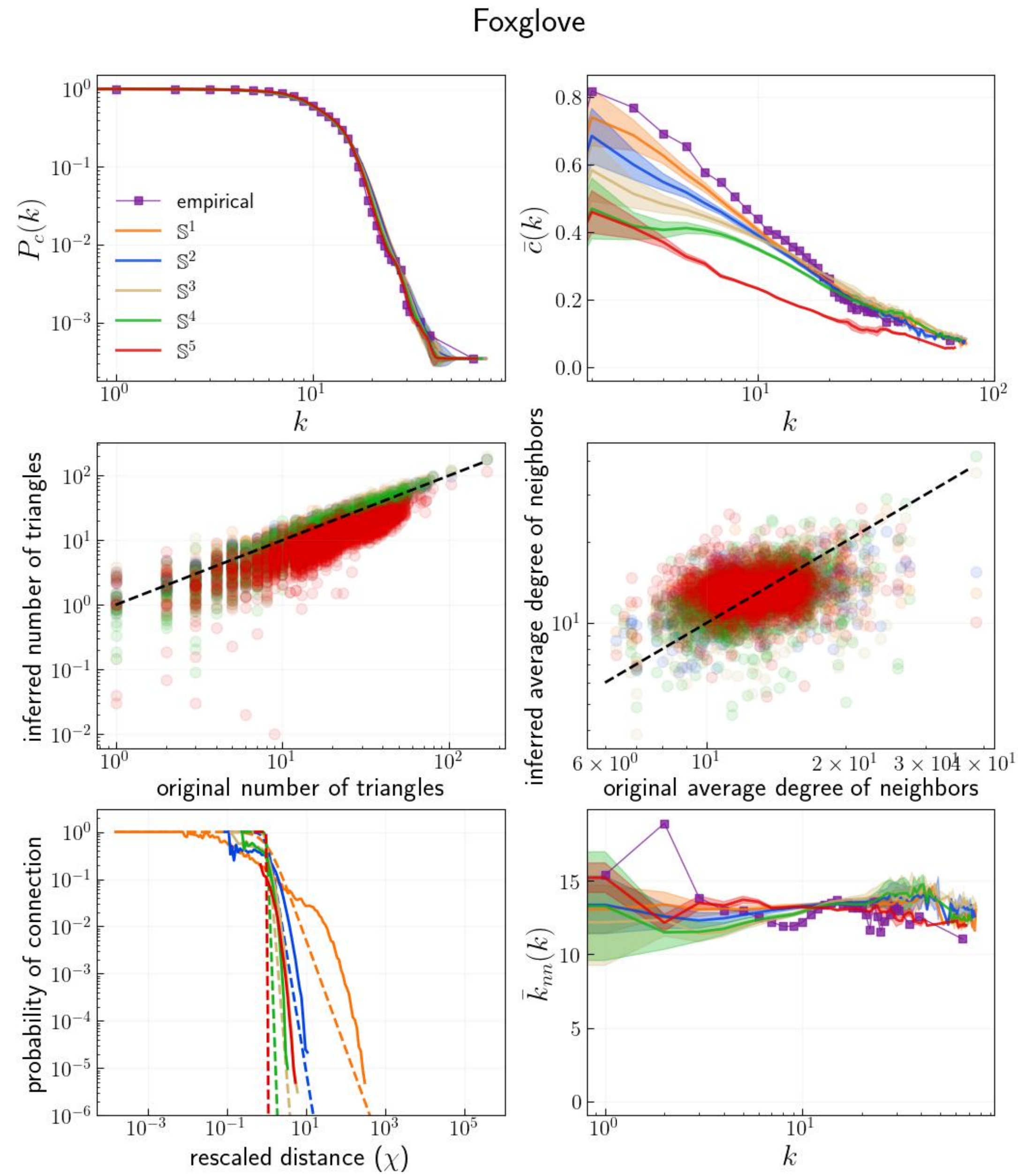

FIG. S31: Topological validation of the embeddings of the Foxglove network. See caption in Fig. S27 for more details.

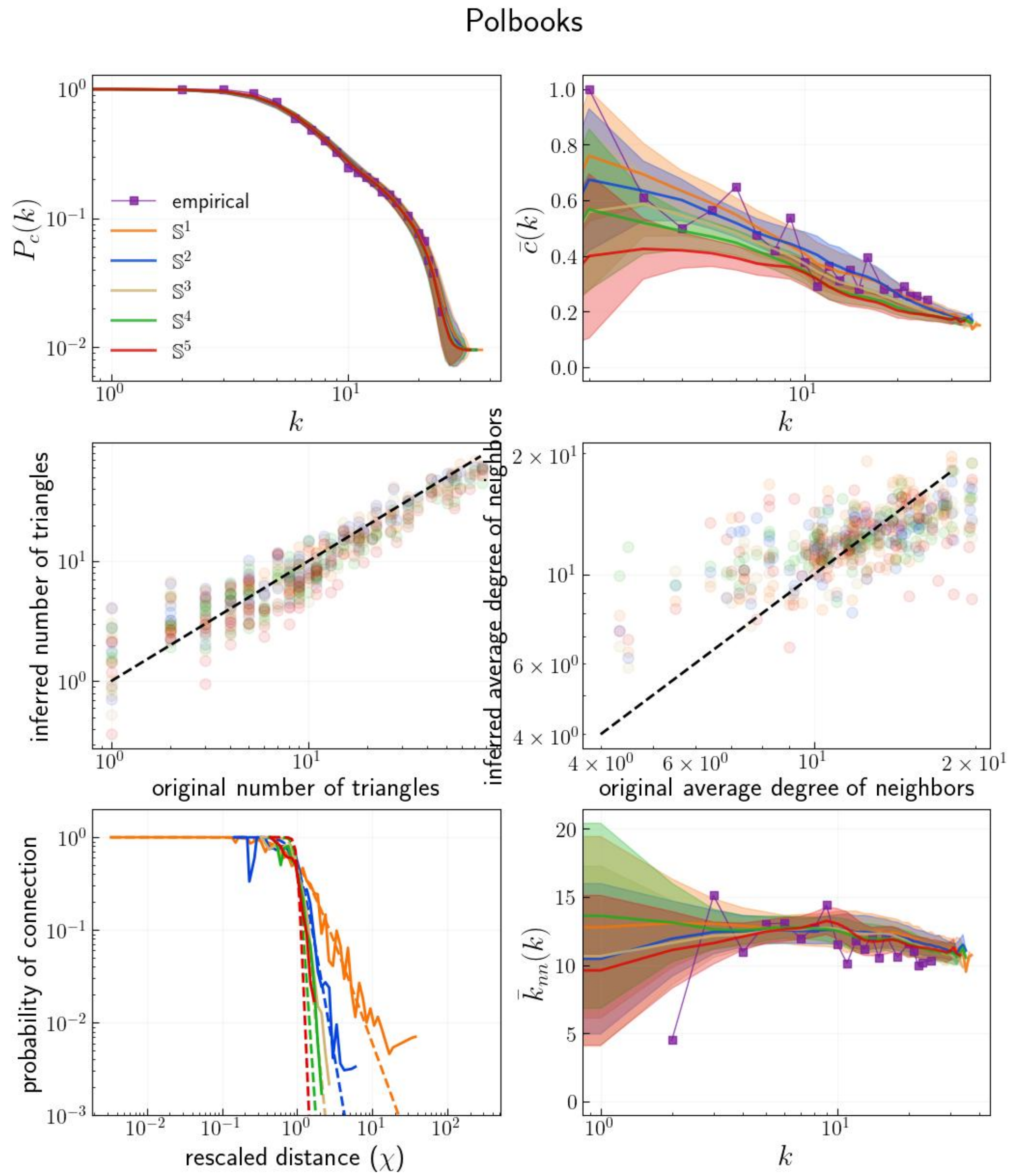

FIG. S32: Topological validation of the embeddings of the Polbooks network. See caption in Fig. S27 for more details.

XII. GEOMETRIC CONCENTRATION OF REAL NETWORKS

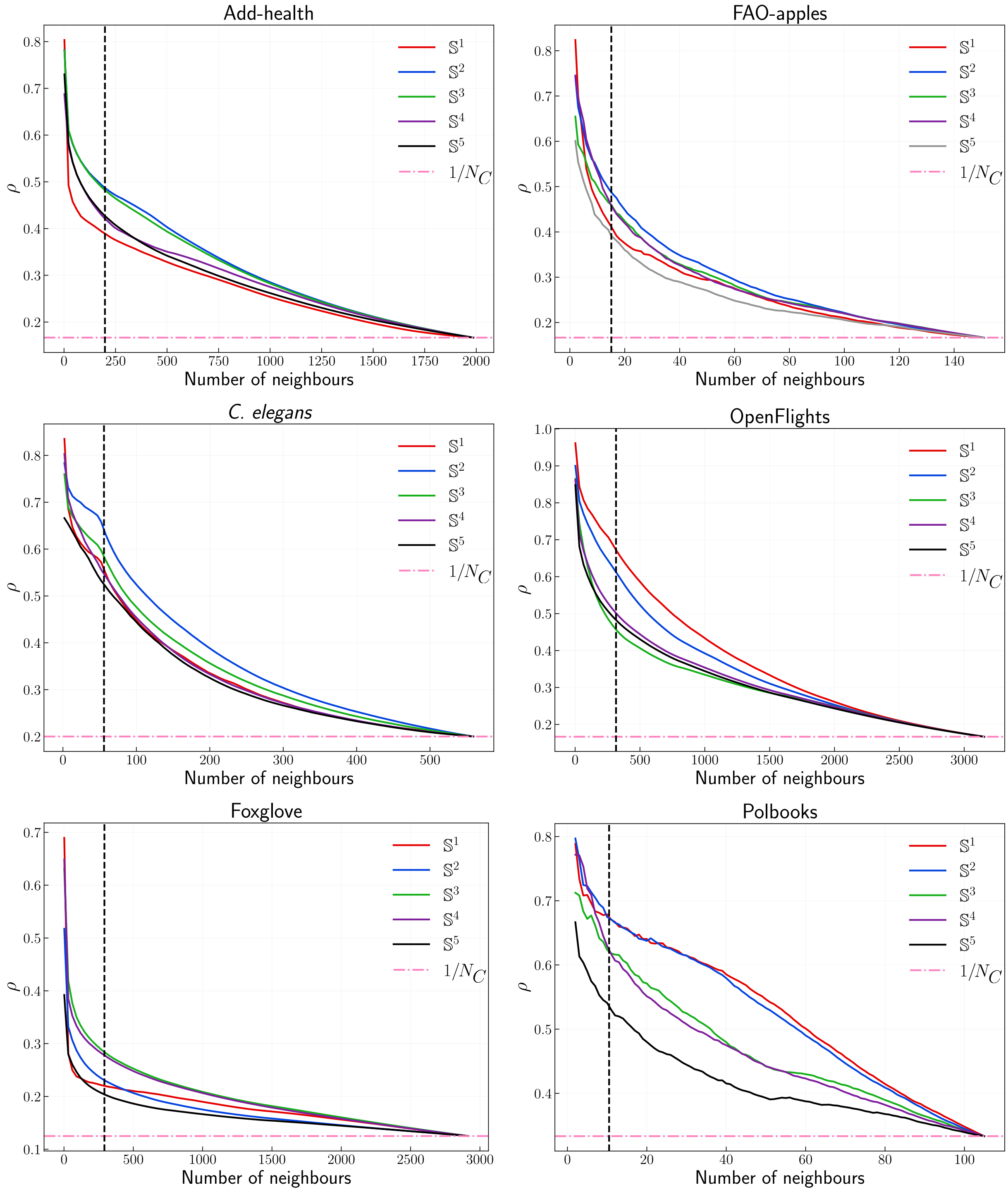

FIG. S33: Geometric concentration as a function of the number of geometric closest neighbours ($n_{i,g}$) for the real networks. Results are averaged over all communities. The pink line indicates the minimum value $1/N_C$ where N_C is the number of communities. The black vertical line represents the $0.1 \cdot N$ threshold at which the community concentration was computed, i.e., $c_C = \rho(n_{i,g} = 0.1 \cdot N)$ and used to compare embeddings in different dimensions.

XIII. COMPARISON WITH TOPOLOGICAL-BASED COMMUNITY DETECTION METHODS

We compare the agglomerative clustering algorithm, which uses the obtained embedding in the best dimension, with the topological-based methods for the community detection task. Table S2 shows the obtained values of modularity, whereas Table S3 shows the Normalized Mutual Information between the predicted communities and the metadata labels. Table S4 depicts the overlap between clusters obtained using the agglomerative clustering algorithm and the four topological-based methods. The number of clusters obtained by each method is reported in Table S5.

TABLE S2: Comparison of the community detection performance in terms of **modularity** (Q) between the agglomerative clustering algorithm based on the embeddings in the best dimension and the topological based methods: GMM (greedy modularity maximization) [1], Louvain method [2], Infomap [3] and LPA (Label Propagation Algorithm) [4]. For the agglomerative clustering algorithm we report two cases: (i) where the number of clusters is obtained from the metadata and (ii) when the number of clusters is determined by the maximum modularity. The highest value is shown in **blue** and the second highest in **orange**.

	agglomerative clustering	GMM	Louvain	Infomap	LPA
Add-health	0.5788 / **0.5796**	0.5413	**0.6138**	0.4182	0.5015
FAO-apples	0.1555 / 0.2029	**0.2334**	**0.2589**	0.0146	0.0016
C. elegans	0.513 / 0.513	0.4877	**0.5284**	**0.5245**	0.4604
OpenFlights	0.5425 / 0.5659	0.6	**0.6582**	0.5622	**0.6129**
Foxglove	0.6952 / **0.6954**	0.5983	**0.7484**	0.6506	0.5732
Polbooks	0.4954 / 0.5123	0.5019	**0.527**	**0.5259**	0.4817

TABLE S3: Comparison of the community detection performance in terms of **Normalized Mutual Information** (NMI) between the predicted communities and the metadata labels for the agglomerative clustering algorithm based on the embeddings in the best dimension and the topological based methods: GMM (greedy modularity maximization) [1], Louvain method [2], Infomap [3] and LPA (Label Propagation Algorithm) [4]. For the agglomerative clustering algorithm we report two cases: (i) where the number of clusters is obtained from the metadata and (ii) when the number of clusters is determined by the maximum modularity. The highest value is shown in **blue** and the second highest in **orange**.

	agglomerative clustering	GMM	Louvain	Infomap	LPA
Add-health	**0.4854** / **0.4717**	0.322	0.374	0.4141	0.3608
FAO-apples	**0.391** / 0.3218	0.3869	**0.5424**	0.1432	0.024
C. elegans	**0.4938** / 0.4938	0.4146	**0.4653**	0.4446	0.4305
OpenFlights	0.6078 / **0.6218**	0.5957	**0.7294**	0.6168	0.5599
Foxglove	0.3321 / **0.3382**	0.044	**0.3437**	0.2221	0.3130
Polbooks	**0.5666** / 0.5299	0.5308	**0.5901**	0.5288	0.4383

TABLE S4: The overlap between the communities obtained by applying the agglomerative clustering algorithm based on the embeddings in the best dimension and four topological based methods in terms of Normalized Mutual Information (NMI). We report two cases: (i) where the number of clusters is obtained from the metadata and (ii) when the number of clusters is determined by the maximum modularity.

	GMM	Louvain	Infomap	LPA
Add-health	0.4129 / 0.411	0.5346 / 0.5543	0.4496 / 0.4104	0.4162 / 0.4543
FAO-apples	0.3638 / 0.3940	0.516 / 0.4664	0.1295 / 0.1298	0.029 / 0.0254
C. elegans	0.6235 / 0.6235	0.6258 / 0.6258	0.6443 / 0.6443	0.6179 / 0.6179
OpenFlights	0.5999 / 0.6019	0.6363 / 0.6485	0.7227 / 0.7698	0.5451 / 0.5519
Foxglove	0.3626 / 0.3927	0.5478 / 0.5943	0.4684 / 0.4852	0.4913 / 0.5146
Polbooks	0.8616 / 0.7732	0.8107 / 0.8502	0.8225 / 0.8430	0.6649 / 0.7184

TABLE S5: The number of clusters found by community detection methods. For the agglomerative clustering algorithm we report two cases: (i) where the number of clusters is obtained from the metadata and (ii) when the number of clusters is determined by the maximum modularity.

	agglomerative clustering	GMM	Louvain	Infomap	LPA
Add-health	6 / 9	12	10	2	184
FAO-apples	6 / 3	5	4	5	2
C. elegans	5 / 5	5	6	10	4
OpenFlights	6 / 5	41	25	4	4
Foxglove	8 / 10	4	15	7	145
Polbooks	3 / 4	4	5	5	8

SUPPLEMENTARY REFERENCES

[1] A. Clauset, M. E. J. Newman, and C. Moore, Finding community structure in very large networks, Phys. Rev. E **70**, 066111 (2004).

[2] V. D. Blondel, J.-L. Guillaume, R. Lambiotte, and E. Lefebvre, Fast unfolding of communities in large networks, Journal of Statistical Mechanics: Theory and Experiment **2008**, P10008 (2008).

[3] M. Rosvall and C. T. Bergstrom, Maps of random walks on complex networks reveal community structure, Proceedings of the National Academy of Sciences **105**, 1118 (2008), https://www.pnas.org/doi/pdf/10.1073/pnas.0706851105.

[4] U. N. Raghavan, R. Albert, and S. Kumara, Near linear time algorithm to detect community structures in large-scale networks, Phys. Rev. E **76**, 036106 (2007).
